# Diagnostic accuracy of artificial intelligence-assisted radiology assessment of cancer: a systematic review

**DOI:** 10.1093/bjrai/ubaf016

**Published:** 2025-11-13

**Authors:** Dylan Zhao, Thomas Packer, Xiaobo Jie, Muhammad Shahid, Jason Oke, Annette Plüddemann

**Affiliations:** University of Oxford Medical Science Division, John Radcliffe Hospital, Oxford, OX3 9DU, United Kingdom; University of Oxford Medical Science Division, John Radcliffe Hospital, Oxford, OX3 9DU, United Kingdom; University of Oxford Medical Science Division, John Radcliffe Hospital, Oxford, OX3 9DU, United Kingdom; University of Oxford Medical Science Division, John Radcliffe Hospital, Oxford, OX3 9DU, United Kingdom; Abbott Diabetes Care Ltd, Witney, OX29 0YL, United Kingdom; Nuffield Department of Primary Care Health Sciences, University of Oxford, Radcliffe Observatory Quarter, Oxford, OX2 6GG, United Kingdom

**Keywords:** cancer, artificial intelligence, diagnostic accuracy, multi-reader mutlicase

## Abstract

**Objective:**

Perform a systematic review and meta-analysis of studies using multi-reader multi-case (MRMC) study designs for cancer diagnosis with artificial intelligence (AI). Review diagnostic accuracy, study design and reporting.

**Methods:**

A search of several databases between January 1, 2014 and February 28, 2024 was performed. Diagnostic accuracy studies that compared radiologists with and without AI-assistance in cancer diagnostic tasks over all imaging modalities were included. Meta-analysis using Summary Receiver Operating Characteristics (SROC) curves were plotted for pooled sensitivity and specificity. Risk of bias was assessed by using the Quality Assessment of Diagnostic Accuracy Studies-Comparative (QUADAS-C) and the Checklist for Artificial intelligence in Medical Imaging (CLAIM).

**Results:**

Thirty-four studies were included of which 23 were included in meta-analysis. Eight identified cancers on Chest X-rays, 17 on CT, 9 on MRI. Pooled sensitivity and specificity were 0.67 (95%CI 0.58-0.74) and 0.82 (95%CI 0.75-0.88), respectively, for clinicians and 0.79 (95%CI 0.71-0.88) and 0.87 (95%CI 0.82-0.91) for AI-assistance. 17 of 34 studies (50%) had concern of bias with QUADAS-C. CLAIM assessment highlighted reporting issues in several domains of methodology in a proportion of studies.

**Conclusion:**

Artificial intelligence assistance tools may benefit clinician diagnostic performance in cancer diagnosis. Updated reporting guidelines may help to overcome potential methodological limitations to clarify AI’s value in healthcare.

**Advances in knowledge:**

Previous reviews compare AI accuracy alone against a clinician. We focus on MRMC study designs to ass AI use in a clinical environment.

## Introduction

Artificial intelligence (AI) and machine learning (ML) techniques in healthcare have demonstrated improvements in accuracy and sensitivity for image-recognition tasks.^1^ In oncological imaging, AI-based tools are being used for increasingly complex decision-making tasks.^2^ Several algorithms have now been approved by the United States Food and Drug Administration (FDA) for medical use.^3^

Despite suggestions that AI matches or surpasses human expertise in diagnosis of multiple cancer types,^4^ concerns have been raised regarding their clinical utility. Doubts centre around an inability to make flexible decisions^1^ in parallel with increased risk of overdiagnosis^5^ and subsequent overtreatment. Furthermore, these tools are often epistemically opaque,^6^ meaning they cannot provide explainable reasoning for diagnoses, essential for deployment in healthcare. AI-assistance could help improve performance of radiologists whilst compensating for limitations of AI-diagnosis alone. Further evidence is required to assess the benefits to routine care. Studies comparing AI models alone directly with radiologist- or clinician-mediated diagnosis have limited clinical utility and thus studies have shifted to implementing a multi-reader multi-case (MRMC) design.^7^ This study design requires multiple radiologists to view cases with and without AI-radiological tools, providing measures of accuracy outcomes and a diagnostic accuracy comparison.^8^

This systematic review assesses MRMC studies using diagnostic accuracy measures to consider whether AI-assisted clinician diagnosis may be beneficial in cancer diagnosis. We describe study design, analysis, and reporting of MRMC studies regarding AI to consider how these may influence the data that is presented.

## Methods

### Protocol and registration

This systematic review was prospectively registered on the Open Science Framework^9^ (OSF) (10.17605/OSF.IO/PWZ9X) (Appendix A1). The study was prepared using guidelines from the Preferred Reporting Items for a Review and Meta-Analysis of Diagnostic Test Accuracy Studies (PRISMA).^10^^,^^11^

### Search strategy and study selection

A search was performed to identify studies that utilized AI tools to assist radiologists in cancer diagnosis and risk stratification, compared with accuracy of a radiologist alone. Studies were required to use any diagnostic accuracy measure as an outcome and were thus not excluded if they did not use measures we later used for meta-analysis. Search terms were combined for 4 key concepts: (1) *cancer/neoplasm*, (2) *artificial intelligence,* (3) *diagnostic imaging*, and (4) *diagnostic accuracy*. This was developed with information specialist advice. The full search strategy is available in the Supplementary Material (Appendix A2). The following electronic databases were searched for English language peer reviewed and grey literature between January 1, 2014 and February 28, 2024: Ovid Medline, Ovid Embase, and Cochrane Central. We chose 2014 as the cut-off as the use of AI algorithms in radiology began to exponentially rise in numbers with huge advances in the capabilities.

The following studies were excluded: (1) comparison studies between AI models against clinicians and studies that describe AI models without comparison, thus not following an MRMC study design; (2) studies calculating the effect of AI on a clinicians’ diagnostic accuracy using statistical methodology (3) studies assessing non-radiological techniques (eg, colonoscopies, endoscopies, or histology), radiation therapeutics or treatments; (4) non-human studies or studies using synthesized data; (5) fMRI studies as computer analysis used here is separate from computer vision-based tasks; (6) reviews, commentaries, and other non-primary studies along with single case reports. Duplicates were excluded using Rayyan.

All stages of study selection were performed by 2 independent reviewers and disagreements were resolved by discussion with a third independent reviewer. A list of excluded studies at full-text stage can be found in Appendix A3, with reasons for exclusion.

### Data extraction

Titles and abstracts were screened prior to full-text screening. Data was extracted using a predefined data extraction sheet by a single reviewer and independently checked by a second reviewer. Extracted information covered broad topics of study characteristics, AI model characteristics, study outcome and study methodology. Characteristics not included in the main text can be found in Supplementary File S1 in the OSF (10.17605/OSF.IO/PWZ9X). We extracted diagnostic performance information including calculated true positive, false positive, false negative, and true negative results among clinicians with and without AI assistance. We recorded collective measures from each study: if this was not provided a mean value was taken of all the values that were reported in the individuals.

### Data and statistical analysis

Where reported, AUC values were extracted and used for data analysis. 2 × 2 classification tables were also derived from reported statistics of sensitivity, specificity and calculated prevalence of positive cases given in all papers. Studies where a 2 × 2 table could not be derived or an AUC was not provided were excluded from meta-analysis. We used the “mada” package for R^12^ to compute summary estimates of sensitivity and specificity with 95% CI of humans with or without AI-assistance using a bivariate model. Summary Receiver Operating Characteristics (SROC) curves were plotted to visually represent the summary estimates of sensitivity and specificity with 95% confidence region and the 95% prediction region, referring to confidence areas that sensitivity and specificity of future studies likely fall into. Where standard errors of reported AUC values were not reported they were approximated using the prevalence of positive and negative cases.

We conducted subgroup analysis based on: imaging techniques (CT and MRI); lung cancer with subgroups of imaging type (X-ray and CT). Additionally, for papers with reported sensitivity and specificity with individual reader data we performed further analyses to consider experience levels. This involved dividing paper’s reader population into Low (<5 years), Medium (5-9 years), and High ≥10 years of experience. 2 × 2 contingency tables were again extracted and averaged across different subsets. Studies were excluded if: (1) no individual data was given, (2) all readers fell into one experience group (eg, readers with all Low experience), or (3) if experience levels were not matched to individual reader data.

### Quality assessment

We assessed risk of bias in included studies using the Quality Assessment for Diagnostic Accuracy- Comparison Tool^13^ (QUADAS-C). Along with this, the Checklist for Artificial Intelligence Medical Imaging^14^ (CLAIM); a list of recommendations for the reporting of such studies including classification, image reconstruction, text analysis, and workflow optimization; was used. Full CLAIM of each study can be found in Supplementary File S2 in the OSF (10.17605/OSF.IO/PWZ9X). This provided sufficient detail for analysis of both diagnostic cohort studies and AI-implementation in radiology. This were performed by a single reviewer with partial verification of a random selection of papers by a second reviewer.

## Results

### Literature search and screening

**Figure 1.**
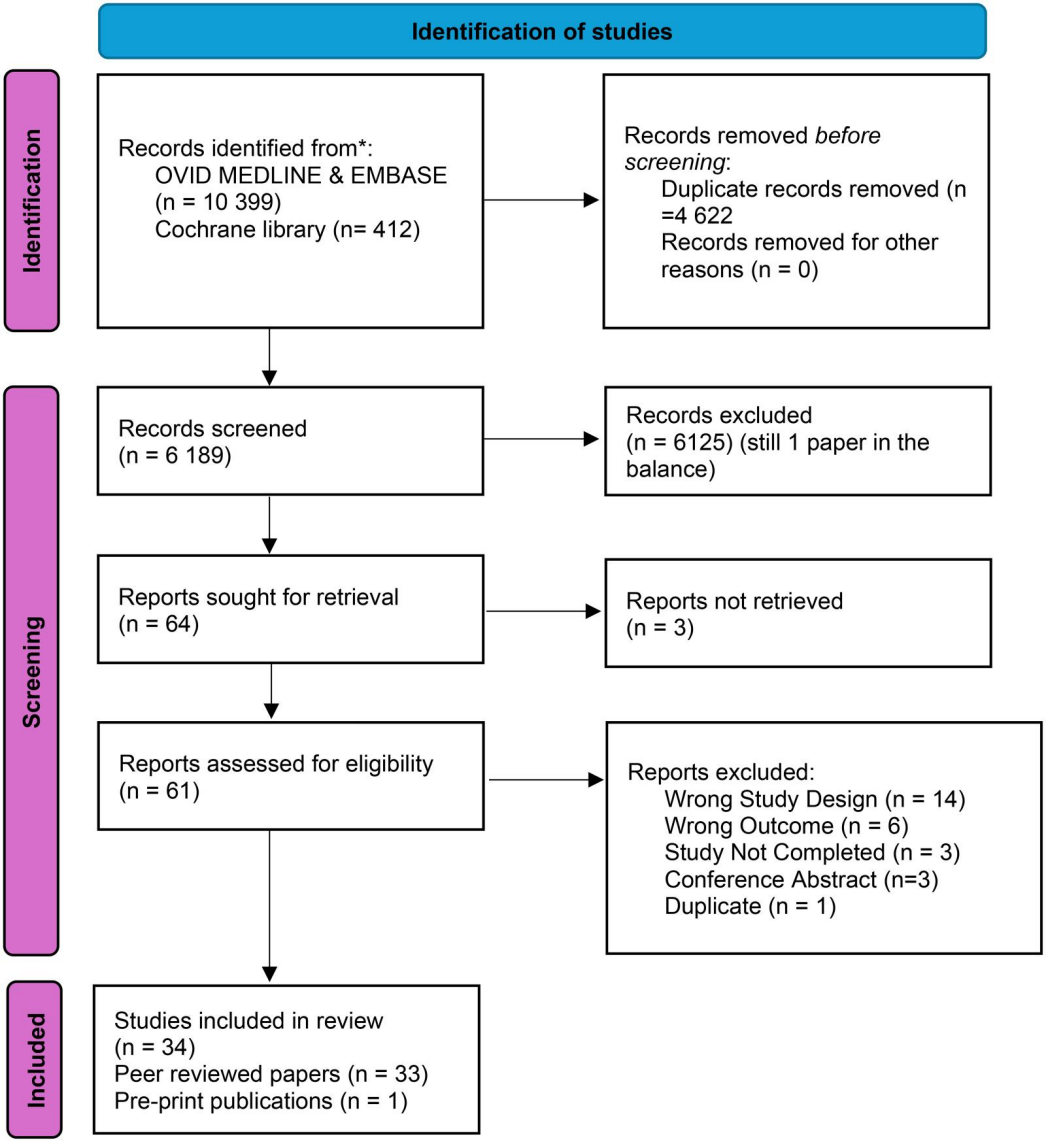
Preferred reporting items for systematic reviews and meta-analyses flowchart showing studies selected for review.

We identified 10 811 peer-reviewed studies, of which 4622 were duplicates. 6125 articles failed to meet inclusion criteria and were excluded. Sixty-four studies were selected for full-text screen of which 34 studies were included in the final review. Thirty-three were peer-reviewed publications and one was a preprint publication (Figure 1).

### Study characteristics

Table 1 presents the characteristics of included studies. Sixteen studies were conducted in lung cancer patients (16/34 = 47.1%) of which 8 studies^15^^,^^22^^,^^25^^,^^31^^,^^35^^,^^37^^,^^38^^,^^45^ (8/16 = 50%) used chest radiographs, 7^18^^,^^19^^,^^21^^,^^23^^,^^26^^,^^27^^,^^40^ (7/16 = 43.8%) computed tomography (CT), and one^33^ MRIs to assess lung and colorectal cancer. Ten other studies (10/34 = 29.4%) used CT to investigate different cancer types (Parotid,^34^^,^^46^ Oesophagus,^36^^,^^42^ Pancreas,^17^ Colon,^48^ Breast,^43^ Adrenal,^16^ Ovarian,^24^ and Nasal/Sino-nasal^30^), while 8 used MRI (8/34 = 23.5%) (Musculoskeletal,^47^ Breast,^44^ Prostate,^28^^,^^29^ Endometrial,^41^ Brain,^20^ Nasopharynx,^32^ Ovarian^39^).

**Table 1.** Included studies, study design and setting, and population characteristics.

First author	Year	Data sources	Imaging modality	Target condition	Diagnostic task	Study randomization or washout	Reference standard
Ajmera^15^	2022	Outpatient & inpatient setting, India	Chest X-Ray	Lung	High/ Low confidence or no nodule	1-month washout	Senior radiologist consensus
Alimu^16^	2023	Patients undergoing adrenalectomy, China	CT (Contrast Enhanced)	Adrenal	Diagnosis	4-week washout	Postoperative histopathology
Anai^17^	2022	Patients undergoing abdominal CTs at hospital, Japan	CT	Pancreas	Differentiate autoimmune pancreatitis and pancreatic duct carcinoma	>1-month washout	International diagnostic criteria with pathology
Chae^18^	2020	Chest CT scans at hospital, South Korea	CT	Lung	Probability of malignancy on 4-point scale	Unclear	Unclear
Chao^19^	2023	Historical chest imaging records, Taiwan	CT	Lung	Lesion identification with a 100-point level of suspicion	4-week washout, index test reversal	Expert panel consensus
Gao^20^	2022	MRI data of patients with brain tumours, China	MRI	Brain	Diagnosis of tumour type	Data randomization	Neuroradiologist consensus
Hempel^21^	2022	Image database from hospitals, Japan	CT	Lung	Appropriate management recommendation	6-month washout	Consensus meeting
Homayounieh^22^	2021	Ambulatory health care centre, Germany, and the Lung Image Database Consortium, US	Chest X-Ray	Lung	Lesion identification and recording presence of 5 findings and level of confidence as well as location	1-month washout	Experienced radiologist consensus
Hsu^23^	2021	Searching electronic medical records database, Taiwan	CT	Lung	Lung nodule identification	6-weeks washout	Senior radiologist consensus
Jan^24^	2023	Suspected ovarian tumours from hospital, Taiwan	CT	Ovaries	CT interpretation and recording each tumour as benign or malignant with other information such as CA125 given.	1-month washout	Unclear
Jang^25^	2020	All patients diagnosed with lung cancer from hospital, South Korea	Chest X-Ray	Lung	Detection with a 100-point level and recommendation for CT follow-up	4-week interval	Diagnosis made based on diagnosis at the time
Kim^26^	2022	Screening and diagnostic chest CT scans, UK and US sources	CT	Lung	Malignancy risk with 100-point level and management recommendation	No washout. Modify with model predictions	Histopathological diagnosis
Kozuka^27^	2020	Suspected lung cancer in CT scans from hospital, Japan	CT	Lung	Pulmonary nodule detection through marking and annotation	14-day interval	Panel of experienced radiologist diagnosis
Li^28^	2021	Patients undergoing prostate mpMRI from hospital, China	MRI (Multiparametric)	Prostate	Cancer likelihood in suspicious area with PI-RADs scores used.	Unclear	Pathological diagnosis
Mehralivand^29^	2020	Patients from multi-institutional dataset	MRI	Prostate	Prostate cancer detection with PI-RADSv2 category	4-week washout	Final histopathological results from prostatectomy
Nakagawa^30^	2022	Patients treated at hospital from with malignant nasal or sinonasal tumour, Japan	CT	Nasal/ Sinonasal	Invasion positive or negative determination	2-months washout	Senior radiologist consensus
Nam^31^	2022	All individuals with chest X-ray for health check-up, South Korea	Chest X-Ray	Lung	Lesion identification with 4-point scale	4-weeks washout	Single senior radiologist diagnosis
Ouyang^32^	2023	Patients with nasopharyngeal carcinoma who had achieved complete remission, China	MRI	Nasopharynx	Detection of local recurrence of cancer and diagnosis	Unclear	Unclear
Rockall^33^	2022	Scans acquired at 16 recruitment sites	MRI	Lung or Colon	Lesion detection via a trained scribe who filled case report forms	4-week washout	Streamline Study Consensus
Shen^34^	2023	Patients treated with PGTs retrieved and reviewed, China	CT	Parotid Gland	Diagnosis with positive and negative labels for benign and malignant respectively	6-months washout	Use of WHO Criteria
Sim^35^	2020	Radiographs obtained in 4 tertiary hospitals, Germany, US and South Korea	Chest X-Ray	Lung	Malignant pulmonary nodule detection with region of interested marked	2-6 hours after initial interpretation	Senior radiologist consensus
Sui^36^	2021	Patients with oesophageal cancer who underwent enhanced chest CT, China	CT	Oesophagus	CT read and cancer diagnosis	30-day washout	Pathological diagnosis
Tang^37^	2023	Text-based search of chest radiograph, chest CT and pathology, Australia	Chest X-Ray and CT reports	Lung	Nodule presence and identification	4-week washout but each set randomly assigned	Criteria for both positive and negative cases
Ueda^38^	2021	Patients who had been subsequently surgically diagnosed with lung cancer, Japan	Chest X-Ray	Lung	Lung cancer nodule detection and annotating bound box on area where lesion present	Unclear	Annotating radiologist consensus
Wang^39^	2021	Patients selected from benign and malignant ovarian tumours, US	MRI	Ovaries	Interpretation of ovary tumour and differentiation	1-month washout	WHO guidelines pathological diagnosis
Wataya^40^	2023	Chest CT examinations at hospital, Japan	CT	Lung	Pulmonary nodule characterization and diagnosis. Evaluated likelihood of presence of 15 characteristics on continuous scales	>1-week after first session	Senior radiologist consensus
Yan^41^	2020	Patients with endometrial cancer who had preoperative MRI from all centres, China	MRI	Endometrial	Identification of pelvic lymph node metastasis	30-day washout	Definite morphological abnormality identification
Yasaka^42^	2023	Patients with oesophageal cancer using the picture archiving and communication system, Japan	CT	Oesophagus	Identification of cancer and malignant potential with a 3-point scale	No washout. Modify with model predictions.	Upper GI endoscopy and histopathological report
Yasaka^43^	2023	Patients with histopathological confirmed cancer and patients without breast cancer between, Japan	CT	Breast	Malignant lesion detection and confidence score diagnostically from 4 being present to 1 being absent.	No washout. Modify with model predictions	Histopathological report and consensus reading
Yin^44^	2023	Breast MRI examinations from hospital, Japan	MRI (Multiparametric)	Breast	Differentiation between TNBC and fibroadenoma	6-weeks washout	Pathological diagnosis
Yoo^45^	2020	Selection of chest X-rays from NLST, a multicentre randomized clinical trial comparing low-dose CT with CXRs for lung cancer screening, South Korea	Chest X-Ray	Lung	Visible lung cancer detection with marking and annotation for suspicious lung cancer.	> 4-week washout period	Senior radiologist consensus
Yu^46^	2023	Patients with histopathologically confirmed BPTs and MPTs from 2 centres, China	CT (Arterial Phase)	Parotid Gland	Assessment of benign or malignant	1-week washout	Histopathological diagnosis
Zhao^47^	2021	Patients diagnosed with musculoskeletal tumours and treated in their institution, China	MRI (Contrast enhanced)	Musculoskeletal	Malignancy determination	1-month washout	Pathological diagnosis
Ziegelmayer^48^	2023	Patients who underwent surgery for colon carcinoma or acute diverticulitis identified, Germany	CT	Colon	Classification as either colon carcinoma or acute diverticulitis	No washout. Modify with model predictions	Histopathological evidence

Varying methodology was used to reduce advantaging comparative index tests through re-reading bias. Two studies^20^^,^^37^ used randomization to prevent visualization of the same data in first and second index tests. Four studies did not include any washout period,^26^^,^^42^^,^^43^^,^^48^ and instead after first review, allowed readers to review their initial answer with AI-input and modify their answers with this new information. Four studies^18^^,^^28^^,^^32^^,^^38^ did not report prevention of re-reading bias. The remaining studies used a washout period ranging from 1 week to 6 months.

Studies also differed in identified ground truths or reference standards. One study used a single person with senior experience,^31^ while twelve studies used group consensus to mitigate interrater variability.^15^^,^^20^^,^^21^^,^^22^^,^^23^^,^^27^^,^^30^^,^^35^^,^^40^^,^^45^ Due to their retrospective nature, others utilized histopathological and clinical diagnoses made at the time.^16^^,^^25^^,^^26^^,^^33^^,^^39^^,^^41^^,^^47^

### Study participants and image population

The number of readers presented by each data set ranged widely (range: 2-18, median: 5.5, IQR: 4-10) (Table 2). The level of clinical experience was reported in all studies except one^33^ which referred to experienced and inexperienced readers. Nineteen studies additionally provided individual quantitative measures (years) of experience.^16^^,^^17^^,^^20^^,^^22^^,^^26–28^^,^^30^^,^^35^^,^^37–39^^,^^41–47^ The mean years of experience per study varied (9.0 ± 5.8). Radiology residents/Students have been excluded from these calculations but were featured in 7 studies.^16^^,^^18^^,^^22^^,^^25^^,^^35^^,^^37^^,^^45^

**Table 2.** Study participants (readers) and image population characteristics.

First Author (no. readers)	Number of readers	Readers	Years of Experience	No. of images presented to each reader (% total)	Total no. of Images per Set	Proportions of cancerous: non-cancerous
Chest X-ray						
Nam^31^	4	Radiologist 1	35 years	169 (100)	169	118 nodules positive, 51 nodules negative
		Radiologist 2	26 years			
		Radiologists 3 & 4	5 years			
Jang^25^	9	6 Radiologists	1-12 years	95 or 96 (24.9 or 25.2)	381	147 radiographs of 117 patients with lung cancers, 234 radiographs of 234 patients with normal radiographs.
		Radiology Resident 1	3rd year			
		Radiology Resident 2 & 3	2nd year			
Yoo^45^	8	Radiology Residents 1, 2 & 3	2nd year	421 (100)	421	75 positives for visible lung cancer and 346 from cancer-negative patients
		Radiologist 1	5 years			
		Radiologist 2	4 years			
		Radiologist 3	5 years			
		Radiologist 4	14 years			
		Radiologist 5	9 years			
Ueda^38^	18	Reader 1	1 year	312 (100)	312	59 malignant and 253 non-malignant
		Readers 2, 3 & 4	2 years			
		Readers 5, 6 & 7	3 years			
		Readers 8 & 9	5 years			
		Reader 10	4 years			
		Reader 11 & 12	7 years			
		Reader 13	8 years			
		Reader 14	9 years			
		Reader 15	10 years			
		Reader 16	11 years			
		Reader 17	12 years			
		Reader 18	22 years			
Homayounieh^22^	10	Thoracic radiologist 1	35 years	100 (100)	100	50 nodules and 50 controls
		Thoracic radiologist 2	25 years			
		Thoracic radiologist 3	21 years			
		General radiologist 1	3.5 years			
		Genera radiologist 2	2.5 years			
		General radiologist 3	9 years			
		General radiologist 4	3 years			
		Residents 1, 2 & 3	1st year			
Ajmera^15^	11	Physician	5 years general medicine	308 (100)	308	All radiographs reported as suspicious pulmonary nodules
		Emergency medicine consultant	4 years			
		Pulmonologist	5 years			
		Anaesthesiologist	3 years			
		Radiologist, 5 years	5 years			
		5 resident radiologists	NA			
Sim^35^	12	3 Resident radiologists	NA	∼200 (25)	800	50 with normal and 150 with lung cancer from each of 4 participating centres (3:1 patient)
		4 Chest radiologists	5 years			
Tang^37^	10	8 radiology attending physicians	NA	70-140 (50-100)	140	81 positives for nodules, 59 negatives for nodules
		Trainee 1	2nd year			
		Trainee 2	4th year			
		Reader 5	1 year			
		5 Chest Radiologists	>10 year			
CT						
Kozuka^27^	2	Reader A	5 years	120 (100)	120	Random selection from those suspected of lung cancer
		Reader B	1 year			
Anai^17^	4	Radiologist 1	5 years	50 (100)	50	20 autoimmune pancreas and 30 pancreatic duct carcinomas
		Radiologist 2	6 years			
		Radiologist 3	24 years			
		Radiologist 4	30 years			
Yu^46^	2	Junior radiologist	5 years	188 (100)	188	130 benign, 58 malignant
		Senior radiologist	15 years			
Chao^19^	4	Junior radiologists	NA	200 (100)	200	100 confirmed to be pulmonary nodules and 100 confirmed to be typical CT images
Shen^34^	12	12 Junior Doctors	<3 years	28 (100)	28	Benign 22, malignant 6
Yasaka^42^	4	Senior radiologist 1	13 years	50 (100)	50	25 oesophageal cancers positive and 25 negative
		Junior radiologist 1	5 years			
		Junior radiologist 2	3 years			
		Junior radiologist 3	2.25 years			
Hsu^23^	6	Chest radiologist 1	5 years	150 (100)	150	52 did not contain any nodules remaining 98 with at least 1 nodule
		Chest radiologist 2	10 years			
		Chest radiologist 3	25 years			
		Junior radiologists 1, 2 & 3	1-2 years			
Ziegelmayer^48^	10	3 radiology residents	< 3 years	60 (100)	60	Balance of AD and colorectal cancer cases
		4 radiology residents	>= 3years			
		3 board-certified radiologists, 2 specializing in GI imaging	NA			
Yasaka^43^	5	Reader 1	12 years	30 (100)	30	Histopathology confirmed breast cancer and a population of 40 without within test population
		Reader 2	6 years			
		Reader 3	4 years			
		Reader 4	2 years			
Kim^26^	12	Reader 1	19 years	300 (100)	300	50% prevalence of malignancy
		Reader 2	7 years			
		Reader 3	15 years			
		Reader 4	10 years			
		Reader 5	3 years			
		Reader 6	1 year			
		Reader 7	4 years			
		Reader 8	4 years			
		Reader 9	11 years			
		Reader 10	2 years			
		Reader 11	3 years			
		Reader 12	2 years			
Alimu^16^	4	Reader 1	10 years	45 (100)	45	Patients who underwent an adrenalectomy in a clinical centre within year from March 2015 to June 2020
		Reader 2	4 years			
		Reader 3	2 years			
		Reader 4	1 year			
Jan^24^	5	3 radiologists	<10 years	56 (100)	56	37 benign, 19 malignant
		2 radiologists	>10 years			
Wataya^40^	15	5 in L group	<3 years	101 (100)	101	46 benign, 55 malignant
		5 in M group	3-5 years			
		5 in H group	>5 years			
Nakagawa^30^	2	General radiologist 1	6 years	49 (100)	49	25 invasion-positive, 24 invasion-negative
		General radiologist 2	3 years			
Sui^36^	3	3 radiologists	5-7 years	100 (100)	100	48 normal cases and 52 cases of oesophageal cancer
Chae^18^	8	Student 1	3rd year	60 (100)	60	30 benign, 30 malignant
		Student 2	3rd year			
		Physician 1	2 years			
		Physician 2	2 years			
		Resident 1	1 year			
		Resident 2	1 year			
		Thoracic radiologist 1	3-5 years			
		Thoracic radiologist 2	3-5 years			
Hempel^21^	2	Reader 1	15 years	50 (100)	50	Cohort size of 50 patients with varying numbers of nodules
		Reader 2	13 years			
MRI						
Zhao^47^	7	Oncologist A,	26 years	304 (100)	304	212 cancerous, 92 non-cancerous
		Oncologist B	23 years			
		Radiologist C	33 years			
		Orthopaedist D	36 years			
		Orthopaedists E, F & G	19 years			
Yin^44^	4	Junior Radiologist 1	2 years	67 (100)	67	32 TNBCs and 35 fibroadenomas
		Junior Radiologist 2	3 years			
		Senior Radiologist 1	11 years			
		Senior Radiologist 2	17 years			
Li^28^	2	Junior Radiologist	5 years	200 (100)	200	100 prostate cancer, 100 non-prostate cancer
		Senior Radiologist	10 years			
Yan^41^	2	Radiologist 1	5 years	622 (100)	622	64 positive PLMN, 558 negative PLMN
		Radiologist 2	10 years			
Gao^20^	9	Neuroradiologist 1	15 years	∼130 (11.1)	1166	NA
		Neuroradiologist 2	11 years			
		Neuroradiologist 3	13 years			
		Neuroradiologist 4	19 years			
		Neuroradiologist 5	15 years			
		Neuroradiologist 6	30 years			
		Neuroradiologist 7	10 years			
		Neuroradiologist 8	9 years			
		Neuroradiologist 9	24 years			
Ouyang^32^	2	Radiologist 1	7 years	140 (100)	140	72 recurrent NPC and 68 recurrent free NPC
		Radiologist 2	>30 years			
Rockall^33^	25	18 experienced readers	NA	Experienced: 15-16 (8.0- 8.5) Unexperienced: 10-14 (5.3-7.4)	188	117 colon cancer, 11 lung cancer, unclear on status of further 60 images.
		7 inexperienced readers				
Mehralivand^29^	9	3 Low	≤1 year	Mean, 78 (33.1)	236	152 cancerous, 84 controls
		3 Medium	1-3 years			
		3 High	>3 years			
Wang^39^	4	Junior radiologist 1	13 years	53 (100)	53	37 benign and 16 malignant
		Junior radiologist 2	10 years			
		Junior radiologist 3	6 years			
		Junior radiologist 4	5 years			
		Junior radiologist 2	10 years			
		Junior radiologist 3	6 years			
		Junior radiologist 4	5 years			

Image population size ranged widely between studies (range: 28-1166, median: 159.5, IQR: 67.5-303). There was discrepancy as to whether image sets were “enriched” with positive target-cancer cases compared to the general population: proportion of populations with cancer ranged (49.3% ±20.9). Six studies did not present the whole dataset to each radiologist^20^^,^^25^^,^^29^^,^^33^^,^^35^^,^^37^ (Table 2), providing each with a set number in each index test.

### Artificial intelligence assistance method and output

Most reported AI algorithms using a convolutional neural network (CNN) (23/34 = 67.6%). Other models being used included a Support Vector Machine Classifier^17^ and 2 Random Forest classifiers.^29^^,^^41^ Twenty-three studies (23/34 = 67.6%) used commercially available or existing AI tools with changes to suit their requirements (Table 3).

**Table 3.** Artificial intelligence tools characteristics, output and application.

First Author	Type of Algorithm Used	Custom deep learning or standard use	Output to readers
Chest X-Ray			
Nam^31^	Deep Convolutional Neural Network	Commercially available from Lunit INSIGHT^56^	Per radiograph probability value between 0 and 1 and per-pixel localization map overlaid on the input radiographs
Jang^25^	Deep Convolutional Neural network	Commercially available from Lunit, INSIGHT	Colour-coded regions map identifying location of lesions in areas with activation values are 15% or greater and have a probability value of 0-100% indicating probability containing a malignant nodule
Yoo^45^	Deep Convolutional Neural Network	Commercially available from Lunit INSIGHT	Probability of pulmonary nodule or mass on CXRs and a heatmap for lesion detection
Ueda^38^	Encoder-Decoder Network Categorizing Segmentation Technique	Commercially available EIRL Chest X-Ray Lung Nodule (LPIXEL Inc.).	Display of bounding boxes on all areas of suspected cancer in a radiograph
Homayounieh^22^	Convolutional, with early feature extractor followed by discriminator sub-network.	AI Rad Companions Chest X-Ray Algorithm (siemens Healthineers AG)	Localizes lesions and assigns a confidence score on a scale of 1 to 10
Ajmera^15^	2-Feature Pyramid Networks each having an Xception encoder	NA	DxNodule AI screen outputs along with original radiographs given to radiologist
Sim^35^	Deep Convolutional Neural Network	Samsung Auto Lung Nodule Detection. Modified version of ResNet-50	Provides region of interest marks indicating findings interpreted as lung cancers
Tang^37^	Unclear	Commercially available AI algorithm Annalise.ai^57^	3 interfaces presented to radiologist: a) text only output (UI-A) b) combined text and AI confidence score output (UI-B) and c) combined text, AI confidence score and image overlay output (UI-C)
CT			
Kozuka^27^	Faster Region-Based Convolutional Neural Network	InferReadCT Lung developed by Infervision Co. Ltd.	Display marks, density, major axis and volume of detected nodules
Anai^17^	Support Vector Machine (SVM) Classifier	Custom developed	Percentage probability of being either AIP or PD via a bar graph
Yu^46^	Convolutional Neural Network	Commercially available from MobileNet V3	Predicted probability of BPT and MPT from optimal deep learning model provided
Chao^19^	Convolutional Neural Network	AI algorithm detection software, V6 Pulmonary Image Computer-Aided Detection Software, V5-MED-LU01	Marks region of interest for readers of suspected pulmonary nodules. Integrated into their DICOM viewer to read image with marked suspects
Shen^34^	Convolutional neural network	3d DebseBet-121 as classifier^58^	Provided with model’s prediction and taken model results as references or taken no account of them based on clinical judgement
Yasaka^42^	Convolutional Neural Network	Programming Language of Python and Deep Learning Framework of Chainer 4.0.0	Model binary output related to cancer presence (Y/N) prediction
Hsu^23^	Background subtraction algorithm for vessel suppression Following CADe processes lug opacities and detects nodules	ClearReadCT system, standard use from Riverian technologies	Removal of pulmonary vessels and classification of nodules as actionable for radiologist.
Ziegelmayer^48^	Convolutional Neural Network	Open source TensorFlow v 2.4, custom.	Presentation of algorithm prediction allowing to change or keep initial assessment
Yasaka^43^	Convolutional Neural Network	Python 3.9.7 and Tensorflow gpu 2.8.0	Output score of deep learning model for right and left breast presented and allowing reader to modify
Kim^26^	Convolutional Neural Network.	Commercially available LCP-CNN CAD software from Virtual nodule Clinic, Optellum.	LCP score and displays lung cancer prediction score to reader to allow to post-CAD risk determination
*Alimu* ^16^	3D U-net consisting of encoder and decoder. Fed through another 3D U-net for rough segmention Similar encoder-decoder with variational autoencoder for bounding box	NA	Kidney segmentation, tumour and adrenal segmentation and refined segmentation and classification. Heatmaps of tumour regions
Jan^24^	Radiomics and a 3D U-net CNN for feature extraction	NA	Output a probability (0-100%) of malignancy for each tumour
Wataya^40^	Deep-Learning Based system	CAD system attached to SYNAPSE SAI Viewer V1.4 FUJIFILM Corporation	Shows pulmonary nodules/ masses in bounding boxes that then marginate and characterize the nodules
Nakagawa^30^	Convolutional Neural Network Algorithm	Visual Geometry Group 16 model developed at Oxford University	Binary classification of invasion positive or negative test
Sui^36^	Convolutional Neural Network	Modified V-NET architecture named VB-NET	Marking candidate oesophageal cancer areas with green boxes
Chae^18^	Convolutional Neural Network	Based on AlexNet	Malignancy possibility of each nodule reported by averaging percentage of malignancy of each slice
Hempel^21^	NA	CAD system available from Veye Chest v2.15.3	Detects and segments pulmonary nodules and provides information such as nodule composition, diameter, volume and volumetric changes over time.
MRI			
Zhao^47^	Deep Convolutional Neural Network	MRNet construction based on the AlexNet	Mapped onto original MRI and colour coded regions suggesting the importance for MRNet to output a probability
Yin^44^	Deep Convolutional Neural Network	Pretrained ResNet 18 on ImageNet with several modifications	For each of the 3 models if it was a TNBC a point was given with a total of 3 for an AI combination score. This score was given to radiologists
Li^28^	Convolutional neural network	Prostate gland segmentation based on VNET, classification based on DenseNet	Deep learning delineations for prostate cancer by cropping out region of interest and classifying.
Yan^41^	Radiomic features selected using Random Forest Classifier to build model	NA	Prediction results of PLNM given
Gao^20^	2-staged Deep Learning System Segmentation network Classification of identified tumour into 18 classes	NA	Tumour classification
Ouyang^32^	Convolutional neural network with segmentation of local recurrence by a nnU-NET with an encoder-decoder	Custom developed	Probability of recurrence and countour region
Rockall^33^	Deep Convolutional Neural Network with 2-stage strategy	Leveraging an existing CNN algorithm^60^ and DeepMedic architecture	Probability heat maps for lesion enabling overlay on original MRI scan
Mehralivand^29^	Multitask Random Forest, like Hough Forest and Regression Forest^61^	Custom	Pixel based cancer probability maps calculated with maximum for attention map boxes corresponding to regions of high cancer probability
Wang^39^	Convolutional Neural Network	ResNet architecture along with EfficientNet with inputs to both	Probability of malignancy for each layer

Algorithm output to aid readers’ decision-making used 2 main methods: colour-coded regions/heatmapping that provided marked regions of interests with probability values^15^^,^^19^^,^^22^^,^^25^^,^^29^^,^^31^^,^^32^^,^^35^^,^^33^^,^^36^^,^^38^^,^^40^^,^^45^^,^^47^ (15/34 = 44.1%), or solely providing a probability of cancer likelihood^17^^,^^18^^,^^24^^,^^26^^,^^34^^,^^37^^,^^39^^,^^41^^,^^43^^,^^46^^,^^48^ (11/34 = 32.3%). Instead of a probability, 2 studies provided a binary value for cancer presence/positivity^30^^,^^42^^,^^44^ while 5 (5/34 = 14.7%) removed surrounding background and displayed the main characteristics of detected nodules.^16^^,^^21^^,^^23^^,^^27^^,^^28^

### Quality assessment

A specific quality appraisal tool to assess domains relevant for both diagnostic accuracy and clinical AI use was not identified. We used 2 tools to separately cover all the relevant domains namely, QUADAS-C^13^ and CLAIM.^14^

**Figure 2.**
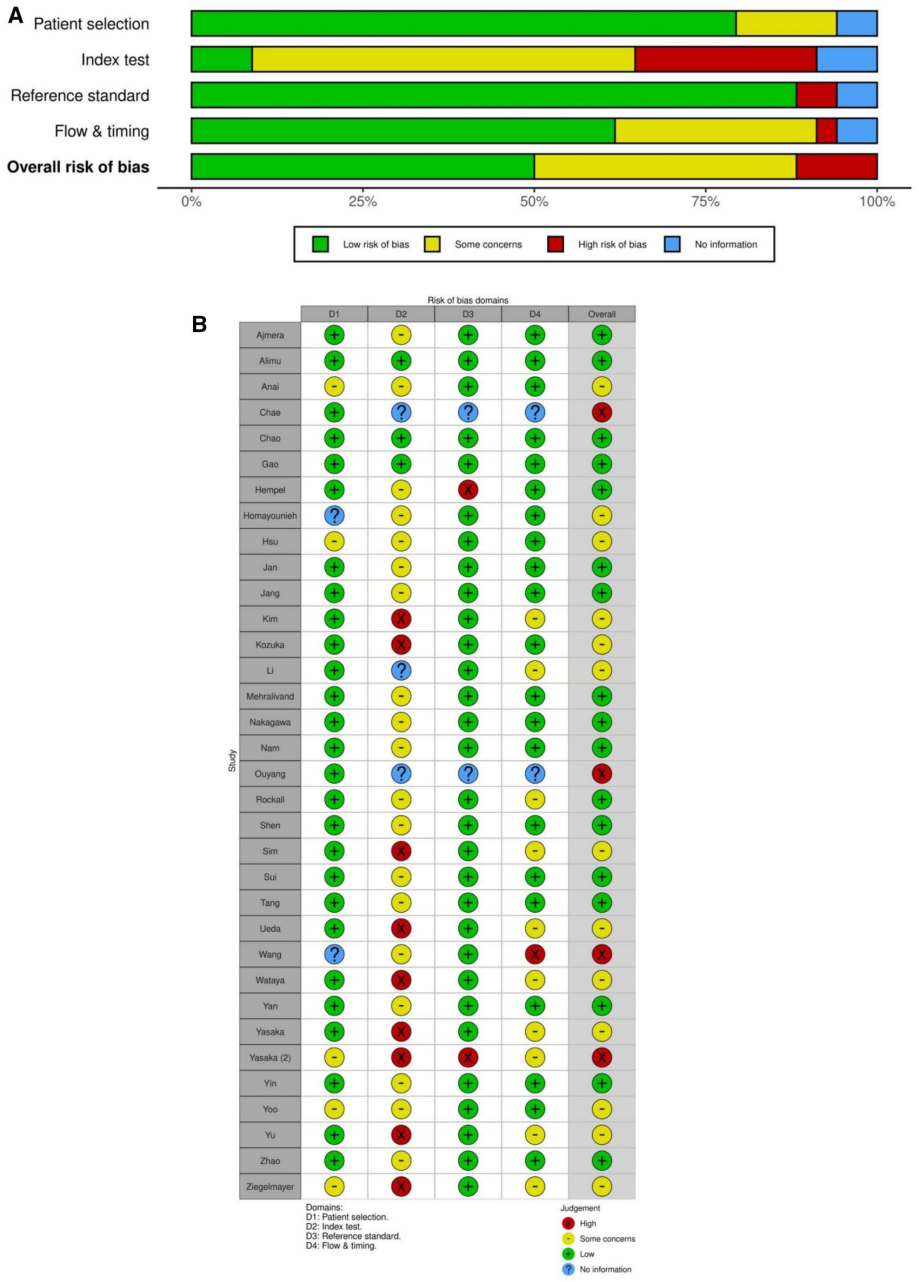
QUADAS-C summary of risk of bias. Shows review authors’ judgement about each domain as (A) percentage of included studies (B) each domain for each included study.

Risk of bias assessment using QUADAS-C highlighted 17 studies as having some concern (13/17 = 76%) or high concern (4/17 = 24%) of bias (Figure 2). Contributing factors mainly revolved around the index test which focused on the order in which index tests were performed. Initial index test interpretation may introduce re-reading bias and influence the second index test, despite having a washout period. For the flow and timing of studies an “appropriate interval” was considered as an interval greater than or equal to one month. This was chosen with consideration of both re-reading bias and the range of washout periods that studies in this systematic review had.

Other domains were reported with generally low risk of bias. For patient selection, the risk of bias overall was generally low as most studies were fully paired and if not, used a random allocation of the image population. Similarly reference standards was generally deemed low risk as most studies did not incorporate the participating clinicians or AI tools into reference standards.

CLAIM, a “best practice” checklist for Medical Imaging with AI studies, was also assessed in all studies. Lower criteria fulfilment was observed in the Methods Section, in particular Ground Truth (0.55 ± 0.31), Data Partitions (0.66 ± 0.33) and Training (0.31 ± 0.32) subsections. The ground truth in the context of an AI model referred mainly to the referenced data elements that would be used for modelling purposes and differs from the reference standard used in the MRMC study design. Furthermore, data partition and training subsections are aimed at AI models for medical imaging as a standalone, focusing on training, cross validation and ensembling techniques used, which are not the focus of our investigated study design.

### Meta-analysis

**Figure 3.**
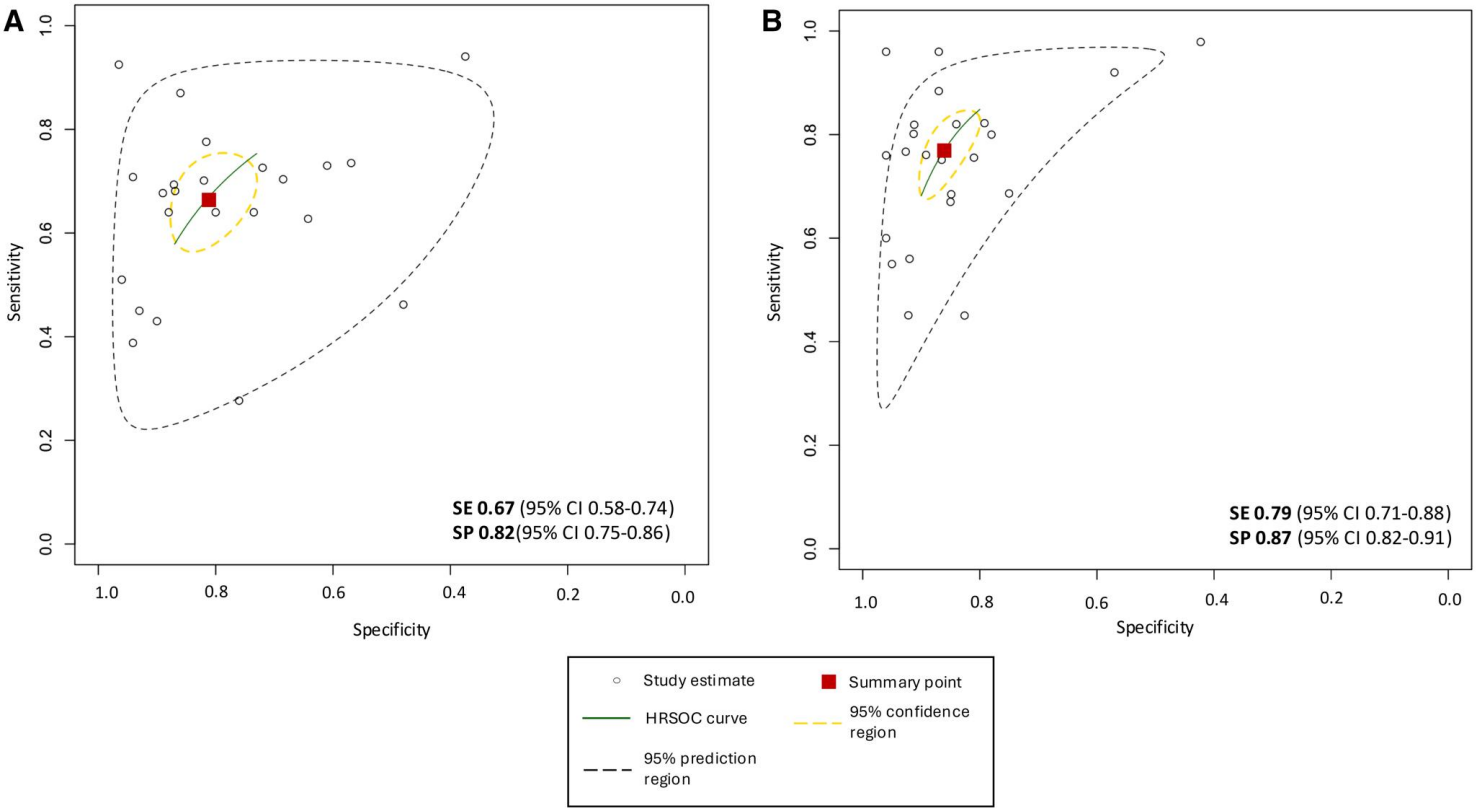
Summary Receiver Operating Characteristics curves. SE sensitivity, SP specificity. Performance of clinicians with (A) no AI assistance and (B) AI assistance. Based on 23 studies, which covered all cancer types and models. The 95% prediction region is a visual representation of between study heterogeneity.

We extracted 2 × 2 contingency tables from 23 of the 34 studies (Supplementary File S3, OSF [10.17605/OSF.IO/PWZ9X]) that were subsequently used for meta-analysis. The SROC curves and forest plots for clinicians without vs with AI assistance are shown in Figures 3 and 4. Sixteen of the 34 studies also reported AUC values that have also been summarized in Figure 5. Overall, clinicians with AI were slightly more accurate than without, but the difference was not statistically significant. For clinicians’ assessment alone, summary estimate of sensitivity was 0.66 (95% CI 0.58-0.74) and specificity, 0.82 (95% CI 0.75-88). Overall diagnostic accuracy increased with AI assistance to a pooled sensitivity and specificity of 0.79 (95% CI 0.71-0.88) and 0.87 (95% CI 0.82-0.91), respectively.

**Figure 4.**
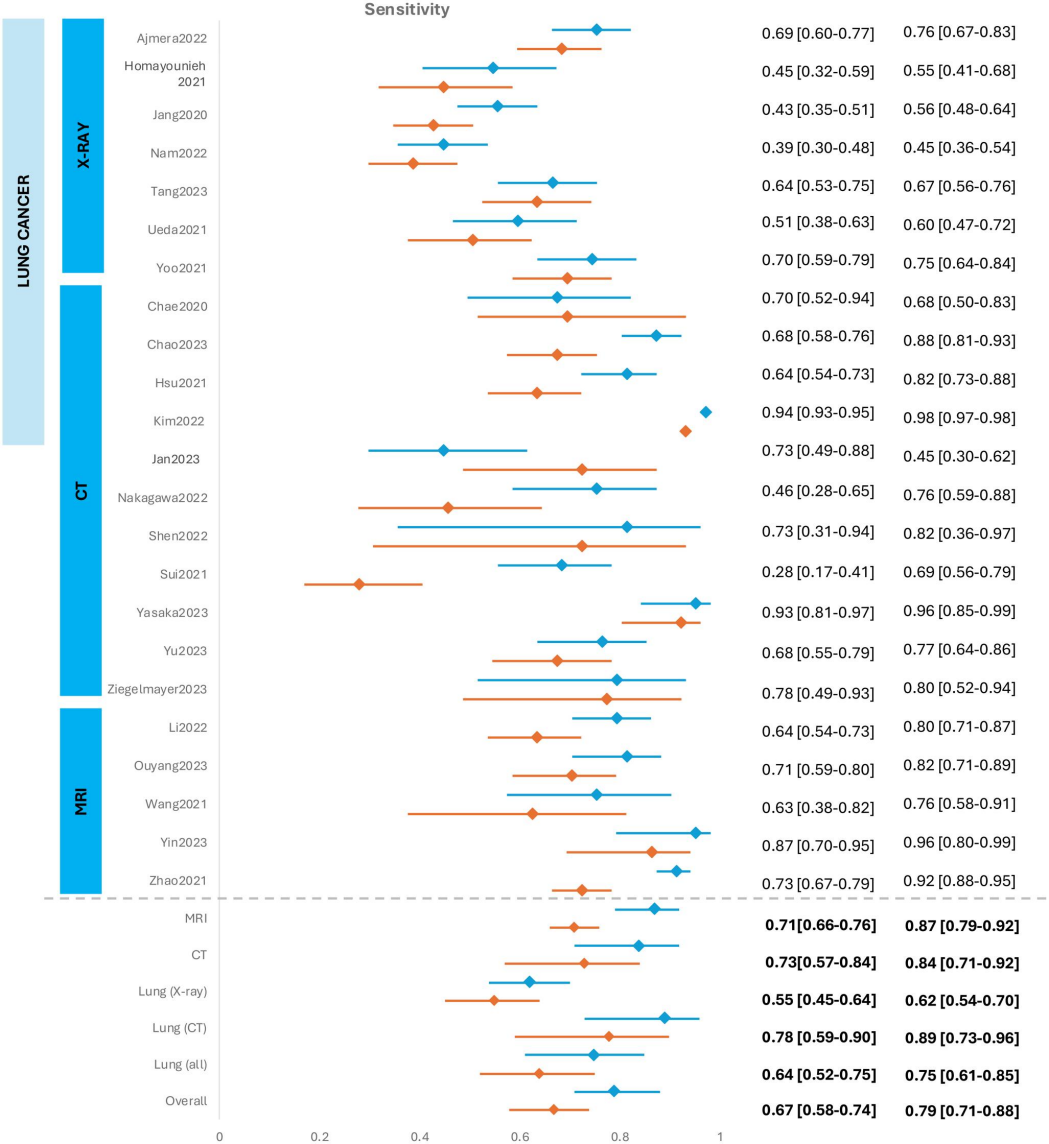
Forest plots of paired sensitivity and specificity. Diagnostic performance of clinicians without (orange) and with AI (blue). Based on 23 studies, which included all models and cancer types. Data is separated into all CT and MRI and lung cancer with subgroup by imaging modality. 2x2 contingency tables, when not present were calculated and rounded to the nearest whole number.

**Figure 4.**
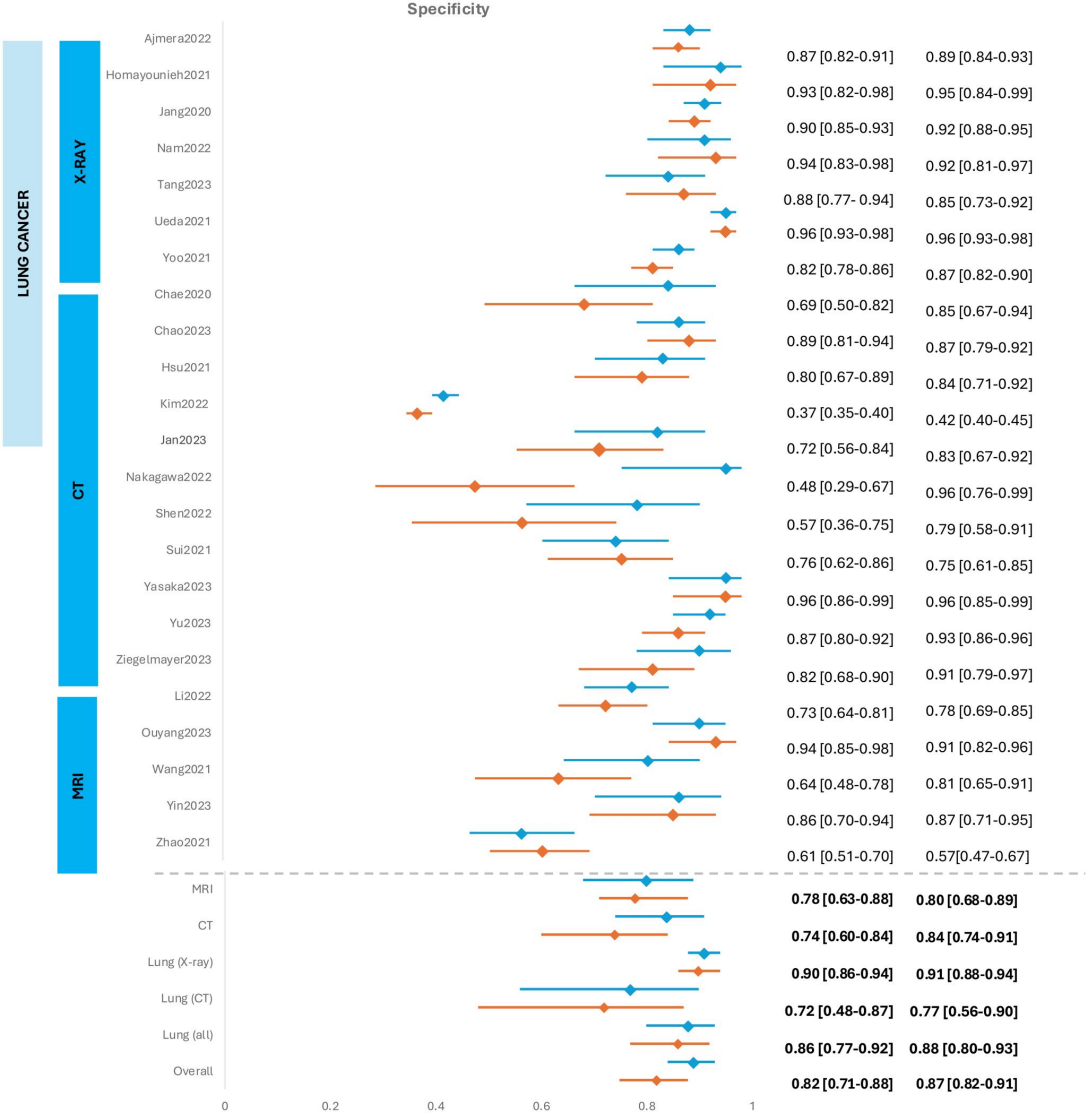
(Continued).

**Figure 5.**
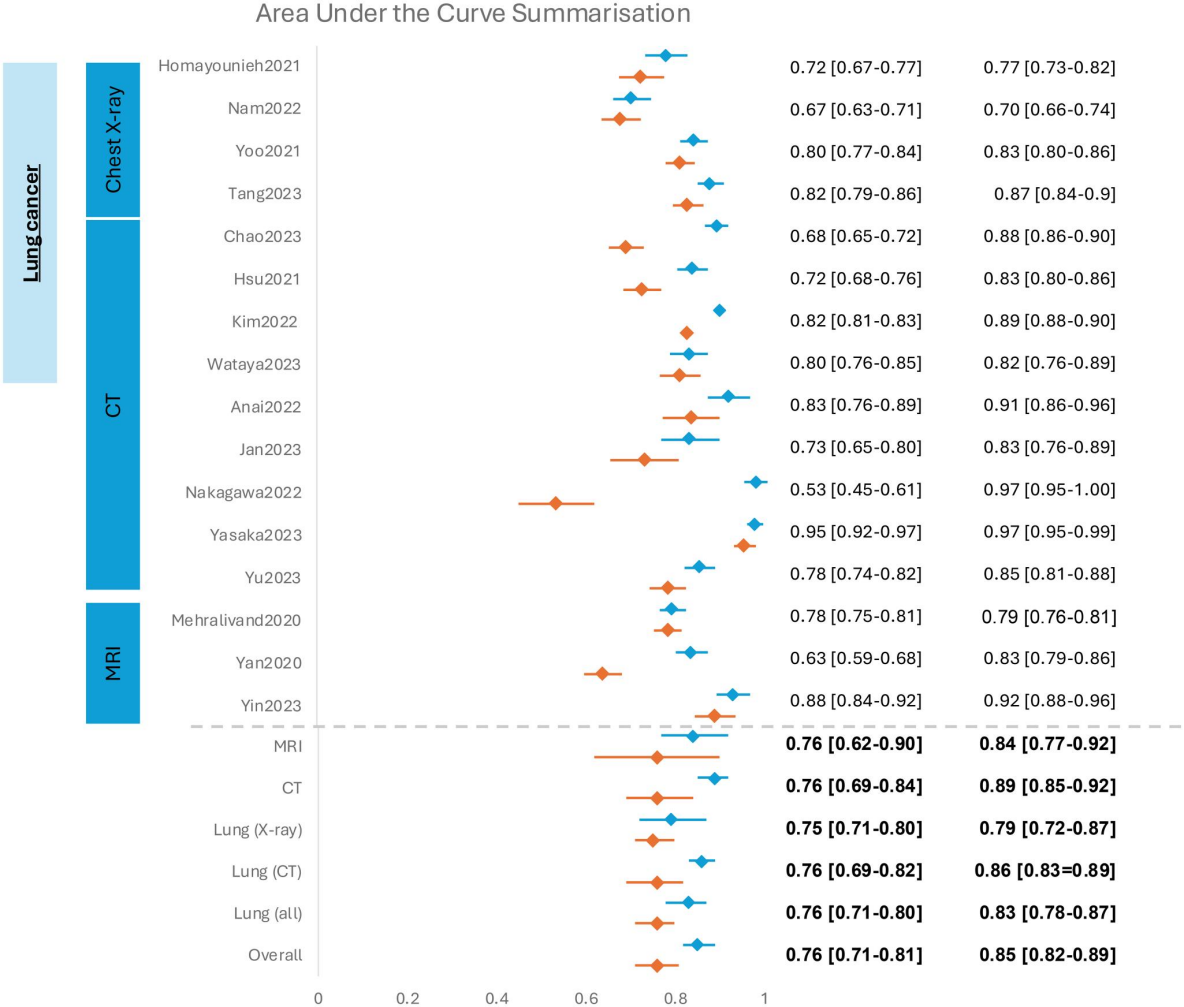
Forest plots of reported AUC among studies. Based on 16 studies which included all models and cancer types. Standard errors when not present were approximated.

The confidence and prediction regions were wide as the studies reported were of a heterogenous nature unified by the MRMC study design in cancer diagnosis. This heterogeneity was multifactorial: (1) different imaging modalities were used over different cancer types, (2) composition of reader groups were of variable size and expertise level, (3) employed methodology varied over studies, and (4) variable thresholds were chosen to evaluate diagnostic accuracy within studies.

Subgroup analysis was performed to adjust for heterogeneity due to cancer type and imaging modality where 4 or more studies meeting the criteria were available (Table 4). Within groups of solely lung cancer with separated distinct imaging modalities there were considerable differences between diagnostic accuracy outcomes in X-Ray and CT scans. CT showed a significantly greater sensitivity compared to X-rays (AI-assisted X-ray 0.62 [95%CI 0.54-0.70] vs CT 0.89 [95%CI 0.73-0.96]) but X-rays had a greater specificity compared to CT (AI-assisted X-0.91 [95%CI 0.88-0.94] vs CT 0.77 [95%CI 0.56-0.90]) (Table 4). MRI studies also exhibited significance in sensitivity increase with AI-assistance (without AI 0.71 [95%CI 0.66-0.76], with AI 0.87 [95% CI 0.79-0.92]).

**Table 4.** Subgroup analysis by cancer type and imaging modality.

Subgroups	Sensitivity (95% CI)		Specificity (95% CI)	
	Clinicians	AI-assisted	Clinicians	AI-assisted
LUNG CANCERS				
**All (*N* = 11)**	0.64 (0.52-0.75)	0.75 (0.61-0.85)	0.86 (0.77-0.92)	0.88 (0.80-0.93)
**CT (*N* = 4/11)**	0.78 (0.59-0.90)	0.89 (0.73-0.96)	0.72 (0.48-0.87)	0.77 (0.56-0.90)
**Chest X-Ray (*N* = 7/11)**	0.55 (0.45-0.64)	0.62 (0.54-0.7)	0.90 (0.86-0.94)	0.91 (0.88-0.94)
ALL CTS				
**(*N* = 11)**	0.73 (0.57-0.84)	0.84 (0.71-0.92)	0.74 (0.6-0.84)	0.84 (0.74-0.91)
ALL MRIS				
**(*N* = 5)**	0.71 (0.66-0.76)	0.87 (0.79-0.92)	0.78 (0.63-0.88)	0.80 (0.68-0.89)
REMOVED NO WASHOUT STUDIES				
**(*N* = 16)**	0.61 (0.53-0.68)	0.74 (0.66-0.82)	0.81 (0.75-0.87)	0.87 (0.82-0.90)
EXPERIENCE LEVELS				
**High (*N* = 10)**	0.77 (0.62-0.87)	0.85 (0.72-0.93)	0.88(0.78-0.93)	0.93 (0.89-0.95)
**Medium (*N* = 6)**	0.63 (0.58-0.68)	0.77 (0.67-0.84)	0.79 (0.62-0.90)	0.89 (0.80-0.94)
**Low (*N* = 7)**	0.62 (0.44-0.77)	0.77 (0.67-0.84)	0.88 (0.73 -0.95)	0.91 (0.86-0.95)

Two outliers^30^^,^^36^ fell significantly below the 95% predicted region for without AI yet were within the region for with AI. We investigated these to elaborate on what factors influenced this. One study^30^ assessed sino-nasal tumour invasions beyond the periorbita, a task undertaken by head and neck radiologists with years of experience. This task was given to 2 general radiologists with no specialization for interpretation of head and neck, meaning a much-reduced expertise levels. With AI input these improved with significant increase in specificity (without AI 0.48 [95%CI 0.29-0.67], with AI 0.96 [95% CI 0.76-0.99]).

Sui et al.^36^ used a population of oesophageal cancer enriched with 48 normal cases and 52 false negatives, initially missed by all radiologists in the hospital with later confirmed pathology. This population represents an extreme in its cancerous cases, which are exceedingly difficult to identify. This also explains the relatively high specificity in this study (without AI 0.76 [95%CI 0.62-0.86], with AI 0.75 [95%CI 0.61-0.85]).

Although there were concerns regarding index test orders and if a washout period prevented re-read bias, there was insufficient data for testing if randomization affected this difference. One of the 2 studies that implemented a randomization process was included in the meta-analysis.^37^ Excluding 7 studies that either did not have a washout period or had no information regarding index test order resulted in changes that were consistent to those seen in the overall pooled data (With AI: Sensitivity 0.61 [95%CI 0.53-0.68], Specificity 0.74 [95%CI 0.66-0.82] and Without AI 0.81 [95% CI 0.75-0.87], Specificity 0.87 [95%CI 0.82-0.90]) (Table 4).

**Figure 6.**
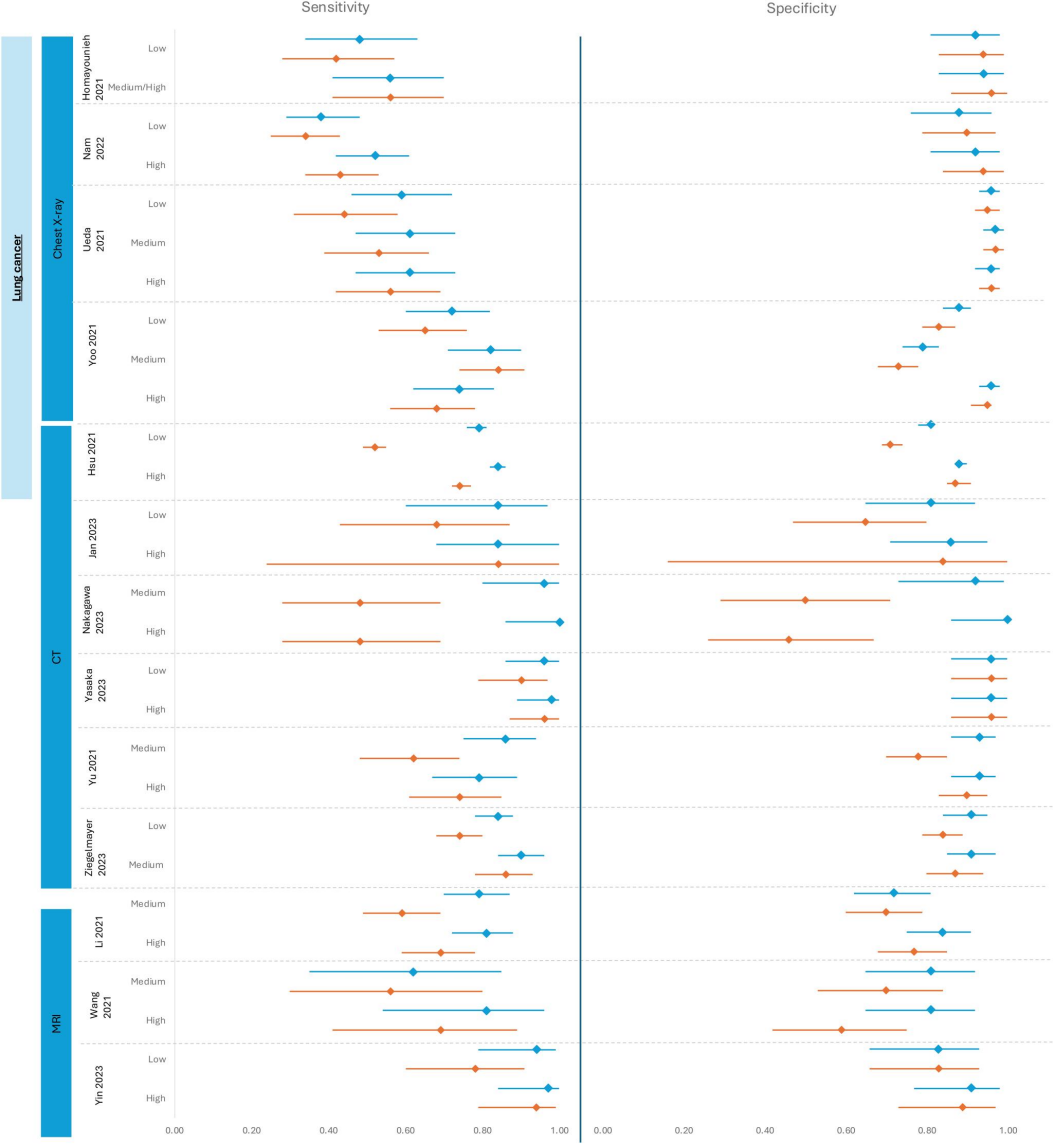

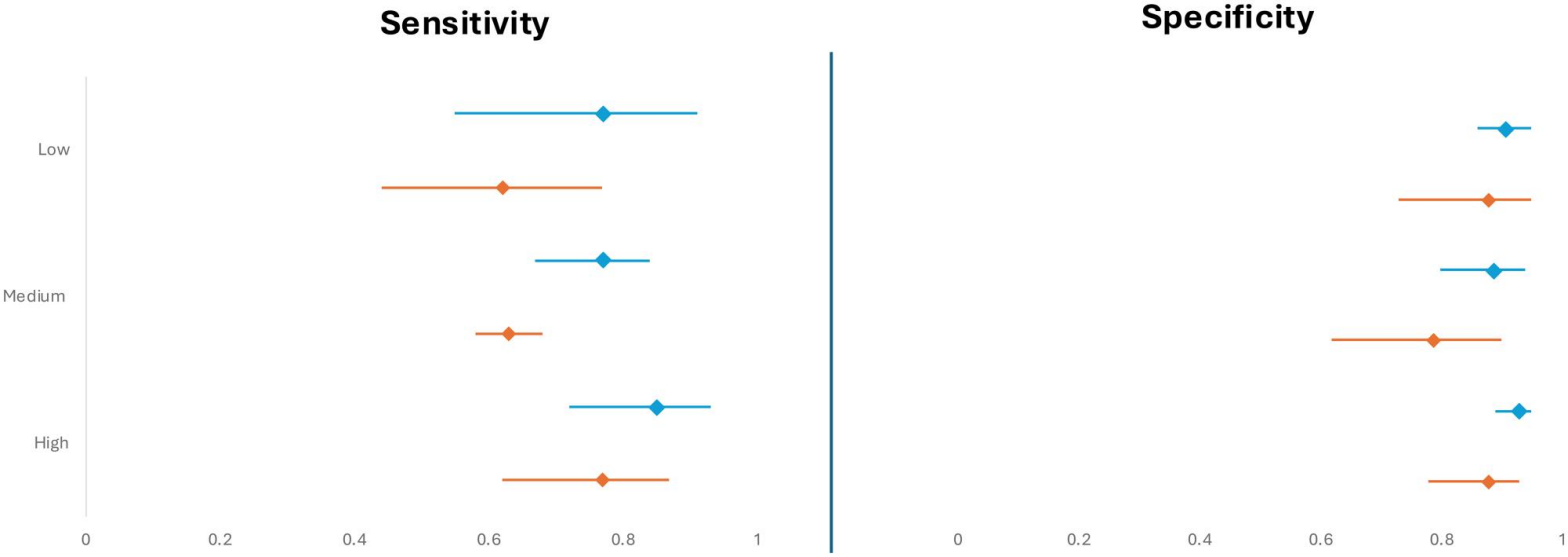
Forest plot comparisons of sensitivity and specificity based on reported experience levels. (A) 13 of the 23 studies where 2 × 2 contingency tables were extracted were included. Experience has been separated into Low (<5 years), Medium (5-9 years) and High (≥10 years). (B) Summary forest plots based on the 13 studies were extracted based on experience.

To determine the importance of experience on AI-assisted improvement, studies where 2 × 2 contingency tables were present were assessed for individual reader data. Thirteen studies reported individual data points that had readers that fulfilled criteria (see Methods) (Figure 6) with summaries for experience levels also shown in Table 4. Grouping by experience meant variable numbers of readers in each study subgroup, with many studies having one reader in a subgroup and thus may confound these results. Overall, several papers showed evidence that lower experienced readers benefited more from AI-assistance. For example, Ueda,^38^ with 18 readers of varying experience for lung nodule detection, showed the sensitivity of those with less than 5 years of experience rose to be of similar standard to those with 10 or greater years. Furthermore, Hsu,^23^ with equal numbers of readers in the low and high-experience group, showed a greater benefit in both sensitivity and specificity for low level readers on computer-aided CT scans for lung nodules.

## Discussion

Most studies of diagnostic accuracy in radiology compare human clinical performance directly against AI alone. This does not consider AI’s benefits in a clinical environment. Investigating how such tools fit into routine care requires standardized study design for direct comparison of clinicians with and without AI. This systematic review and meta-analysis included 34 studies analysing their results, reporting standards, and quality in assessing AI tools as an adjunct in cancer diagnosis.

The results from this systematic review and meta-analysis suggest that AI tools have shown promise in improving diagnostic accuracy for cancer diagnosis. However, the results of our meta-analyses must be taken in the context of the different cancers, imaging modalities and model types utilized, with outcomes showing substantial variation depending on both.

Studies included in meta-analysis failed to explicitly state threshold values with only one study being explicit regarding this information.^26^ Sensitivity and specificity measures vary with given thresholds, and we were unable to account for this in the HSROC model due to this information not being reported in most studies. Therefore, our pooled summary estimates of sensitivity and specificity correspond to an unspecified mix of thresholds and should be interpreted as averages that have been calculated over a range of heterogeneous scenarios. Here the data has been collated to show the effect that AI may have in the radiology setting. Within the context of clinical interpretation, future studies should thus provide more details on a reported threshold and give reasoning as to why the threshold has been used. The values of what thresholds may be used may differ across scenarios in clinical settings depending on factors such as cancer type, imaging modality and for what purpose the radiology is being used. In addition, we have included summarized AUC, which do not rely on thresholds due to the utilized C-statistic: these similarly showed improved diagnostic accuracy with AI tools.

Point estimates for each study, based on calculated differences of diagnostic accuracy measures between paired data would be beneficial in analysis of quantitative improvement between tests. Insufficient data was provided from studies to calculate this metric, which would allow for more formal comparative statistical tests too be performed in addition to comparison of CIs between clinicians alone and AI. Namely, this would require CIs of the differences, which we could not approximate without making large assumptions. Only 2 studies provided this information.^22^^,^^33^

Within-group variation between subgroups of lung cancer by imaging modality were seen. Differences here may depict the difference in resolution between X-Rays and CTs. X-rays are generally used for urgent referral^49^ upon suspicion, due to their relative inexpensiveness and availability. In comparison, CT scans provide a more detailed image, albeit more expensive, that are recommended for diagnosis and staging of disease^50^ and are often used after unclear diagnosis from X-ray.

Reader experience levels were also compared within studies. This showed evidence supporting AI assistance in supporting junior radiologists to bridge the gap with more experienced clinicians. Between study comparison of differences has not been performed as, along with the areas of heterogeneity mentioned above, numbers of readers and the proportions of high, medium and low experience levels varied between studies. To further discern the effects of experience, primary studies should be performed with subgroups with multiple readers of varying expertise.

Methodological approaches may have had a role in the variation in study findings. Many studies did not use any randomization techniques, and several omitted a washout period between the 2 branches of the experiment. This would ultimately lead to concerns of re-reading bias towards the branch that was completed second^51^ which was often AI-assisted. There was insufficient evidence to comment on how randomization may affect outcomes. Furthermore, image populations were often enriched (ie had enhanced number of cancerous cases). Knowing there were more cancerous cases in the image population may affect participant decision-making and would not be representative of a clinical setting. This was identified in Sui,^36^ an outlier study, that solely considered previously unidentified oesophageal cancers. This poses a significantly different challenge than one that would be experienced on a clinician’s day-to-day.

What algorithmic output is relaying to a clinician was an important feature to assess. Several AI tools solely provided a probability or binary value of cancer probability. Clinical viability of these tools is reduced due to a lack of visualization or explainability of the tool’s conclusions. Despite the great potential benefits, ensuring AI gives accurate, reliable and interpretable outputs is vital. A study found that incorrect “sham” AI decisions caused radiologists to make incorrect decisions when initially correct without.^52^ Bias may be introduced due to how information may be presented and thus it is important that AI information is explainable and accurate.

Some limitations need to be considered when interpreting the findings. The search strategy employed in this study was sensitive, aiming to identify all studies that discussed diagnostic accuracy but was not specific. Thus, many studies were screened, and relevant studies may have been missed.

Studies using an MRMC study design still have limited clinical applicability due to methodology queries that may introduce bias in favour of AI tools, as well as enriched populations, unrepresentative of the clinical populus. AI extensions to QUADAS, such as QUADAS-AI,^53^ and other quality assessment tools are currently being developed.^54^ In parallel, guidelines for MRMC study designs should be developed to improve conduct and reporting of such studies. Main foci should revolve around: (1) ease of identification of such studies within the AI and radiology literature, (2) outcomes with clear CIs of the difference between clinicians and clinicians with AI assistance that will benefit future analysis, and (3) addressing the methodological issues mentioned above.

AI tools and methods are tasked with increasingly complex tasks. However, for AI to act as a useful adjunct, clinical studies require collaboration with clinicians providing input on what would aid utility of these tools and efficient workflow in a clinical setting.

## Supplementary Material

ubaf016_Supplementary_Data
