# Supplementary material for: Diagnostic accuracy of artificial intelligence-assisted radiology assessment of cancer: a systematic review
Source: BJR Artif Intell. 2025 Nov 13;2(1):ubaf016. doi: 10.1093/bjrai/ubaf016 (PMC13045702; doi:10.1093/bjrai/ubaf016)

Figure S1: SROC curves using 65% Threshold results.

Kim et al. reference 26 used two different thresholds for sensitivity and specificity. SROCs in Figure 3 were run using the 5% Kim values. Rerun with 65% threshold SROC are shown.

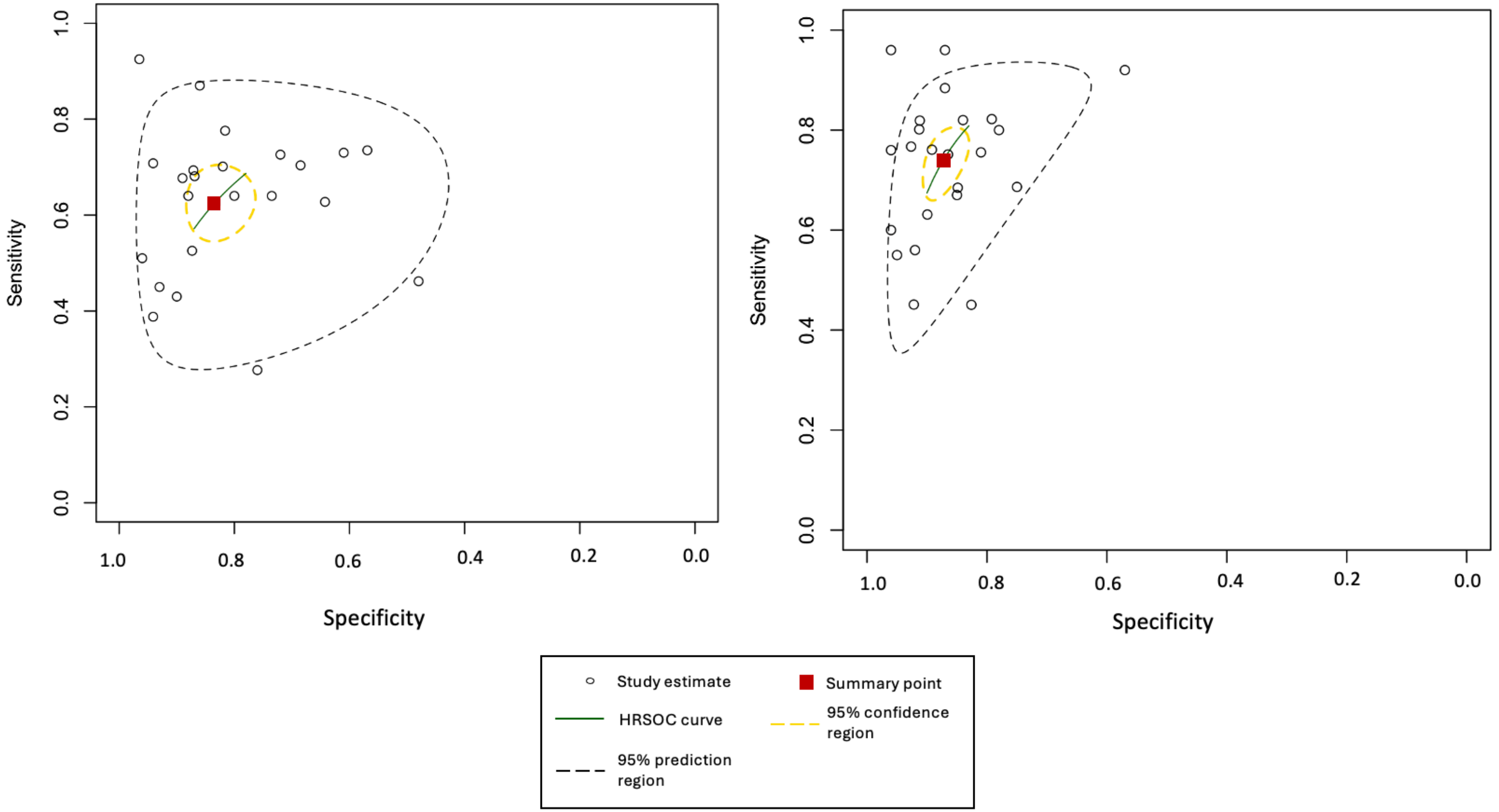

Supplement: ubaf016_Supplementary_Data [file ubaf016_supplementary_data.zip › Figure S1.pdf]
