# Supplementary material for: Diagnostic accuracy of artificial intelligence-assisted radiology assessment of cancer: a systematic review
Source: BJR Artif Intell. 2025 Nov 13;2(1):ubaf016. doi: 10.1093/bjrai/ubaf016 (PMC13045702; doi:10.1093/bjrai/ubaf016)
Supplement: ubaf016_Supplementary_Data [file ubaf016_supplementary_data.zip › Supplementary Material.pdf]

**Appendix A1: Study Protocol**

---

## 1.0 Background

The use of artificial intelligence (AI) and machine learning (ML) techniques in healthcare has become a topic of significant discussion. Methods using such algorithms have demonstrated remarkable improvements in terms of accuracy and sensitivity in medical image recognition tasks[1]. In clinical practice, evidence has shown that AI technology can be used as a clinical decision tool to diagnose numerous diseases when using radiological techniques such as X-ray, CT, MRI, or ultrasound[1]. ML algorithms thus are seen as being able to efficiently provide diagnostically accurate data to patients on a shorter timescale.

**Challenges and complications**

Although AI has been lauded as a revolutionary technique and can be suggested to perform better than humans in healthcare on several issues regarding their accuracy and utility in clinical practice, some of the main issues that have arisen are:

- Algorithms are normally inputted with a specific output in mind while a doctor may be able to make a more flexible decision. For example, an AI tool that has been developed to consider lymph node metastasis in pancreatic ductal adenocarcinoma may be able to outperform radiologists[2]. However, this model will not be able to then make decisions that only a doctor can on the best course of action for the patient.
- **Overdiagnosis**[3]: The algorithms from AI detect and diagnose conditions whether they cause harm for a patient or not. For example, if we consider a CT pulmonary angiography (CTPA) to detect pulmonary emboli (PE) in the lungs, this is usually an incidental finding and even when detected, PE only needs to be treated on a symptomatic basis. AI tools for CTPA may detect this at a much higher rate. Are smaller lesions therefore being detected with the extra sensitivity that are less harmful? The question then arises on how we should approach this data: who should be treated and who should not?

**AI application in medical imaging analysis**

The future of AI seems to be as a diagnostic support tool integrated within a radiologist's workflow, allowing an increase in efficiency and a reduction in errors[1]. Studies that thus compare AI models with radiologist or clinician-mediated diagnosis may not highlight the

effects AI may have in a clinical setting and quantify the gain in accuracy from AI in reading images.

More radiological test studies have thus shifted to implementing a multi-reader multi-case (MRMC) design[4]. This design is popular as once a radiologist has viewed twenty cases there is less information to be gained by asking them to view a further twenty than asking a different radiologist to view the same twenty[5]. The study design asks multiple radiologists to view the same cases and means that results can correlate within readers meaning that bootstrapping and modelling can account for these parameters to enhance statistical power. These studies' primary outcome arises as a receiver operator characteristic (ROC) curve that describes a plot of sensitivity vs. 1-specificity for diagnostic tests[6]. The AUC can then be used as a measure to combine both sensitivity and specificity to compare diagnostic tools.

In recent years, several systematic reviews regarding artificial intelligence and radiology have been published[7,8]. These have focussed on diagnostic performance and how AI models compare to a radiologist. Due to the heterogeneity of data across the field of AI tools these have often been narrative syntheses. By solely considering the MRMC study design this allows examination of a more homogenous dataset. The study design outlined above also will help interpretation of how AI-diagnostic tools may help optimise cancer management in healthcare. Studies using this as a primary outcome are often required by regulatory bodies for licensing new radiological devices[9]. Furthermore, by solely analysing MRMC studies this also will allow consideration of detailed methodological components across studies to consider data presentation and analysis within the field of AI and radiology.

This systematic review thus aims to assess MRMC studies using ROC AUC outcome measures to consider whether AI-assisted clinician diagnosis may be beneficial. The review will also examine design, analysis, and reporting of MRMC studies regarding AI to consider how these may influence the data that is ultimately presented.

## 2.0 Review question

In multi-reader multi-case study designs do AI-diagnostic tools improve outcomes for radiologists in cancer risk stratification?

Population

- Radiological patients analysed for cancerous nodules on radiology

Index test

- Clinicians with computer/AI-aided diagnostic tools

Reference Test

- Clinicians without AI-aided diagnostic tools

Target condition and Outcome

- Diagnostic accuracy/ risk stratification/ determining malignancy

## 3.0 Methods

### 3.1 Study design

We will conduct a systematic review following the Preferred Reporting Items for Systematic Reviews and Meta-Analyses (PRISMA)[10] and Cochrane Collaboration Handbook[11].

### 3.2 Searches

Potential eligible primary radiology AI studies published between 2010-2023 will be identified by searching the following database sets: MEDLINE, EMBASE & COCHRANE

Search Terms: ("Cancer" OR "Tumo*" OR "Malignancy" OR "Neoplas*" OR "Nodule" OR "Carcinoma" OR "Lymphoma")

AND ("AI" OR "Artificial Intelligence" OR "Machine Learning" OR "Algorithm" OR "Computer-assisted" OR "Deep Learning" OR "Radiomics" OR "Neural Network")

AND ("Radio*" OR "X-Ray" OR "PET/CT" OR "MRI" OR "Radiological Images" OR "CT" OR "Computerised Tomography" OR "Magnetic Resonance Imaging" OR "Diagnostic imaging" OR "Image interpretation" OR "tomogra*")

AND ("Sensitivity" OR "Specificity" OR "Diagnos*" OR "Detect*" OR "Accura*" OR "Area Under Curve" OR "Observer variation" OR "Interobserver varia*" OR "Interobserver agreement" OR "Roc" OR "Predictive value" OR "PPV" OR "NPV" OR "auc")

Two authors will independently screen all titles and abstracts for inclusion. The need for additional search terms may be reviewed at this stage. Final search strategy will be then developed with advice from an information specialist and will be included within the appendix of our manuscript for publication. Any deviations from the search strategy stated will be explicitly stated.

Full texts of potentially eligible studies will be assessed independently by two study authors. Disagreements will be resolved by discussion or by consulting a third review author.

### 3.3 Eligibility criteria and study selection

*Inclusion Criteria:*

- Time Frame to search 1st Jan 2014 – 31 September 2023.
    - Papers regarding the use of artificial intelligence/ machine learning in radiology begin to exponentially rise in numbers, with huge advances in the capabilities of these tools in years starting 2014-2015.

  Study types: Multi-reader multi-case test accuracy study
    - These will importantly compare use of AI/ ML/ Computer-aided tools **with** a clinical radiologist/ clinician vs. a radiologist/ clinican alone, in an MRMC study design.
    - Studies will also not be excluded based on publication status.
    - Studies must be published in English.
    - Studies will not be excluded based on whether they use retrospective or prospective datasets.
- Population characteristics for included studies.
    - Medical image data from real patients
    - Imaging used for diagnosis and risk stratification of cancer.
- Diagnostic test type:
    - Studies based on radiographic, computed tomography (CT), magnetic resonance imaging, ultrasound or nuclear medicine/molecular or hybrid imaging techniques.
    - Study must include an AI/ ML tool that is being used in conjunction with radiologist.
    - Radiomics will also be included.
- Outcomes:
    - Attempts to use a MRMC ROC-AUC analysis as a study outcome.
    - Studies must measure diagnostic accuracy as a primary outcome. These should be comparison between clinician with AI and clinician without AI.
    - Study must use a comparative outcome to allow for measurement of change in accuracy when an AI tool is added.

*Exclusion Criteria:*

- Comparison studies between AI models **alone** and clinicians.
- Non-radiological techniques- e.g., colonoscopies, endoscopies or histology that may arise in malignancy diagnosis.
- Studies that relate to radiation therapy or using radiological techniques in cancer treatment
- Studies that are monitoring for recurrence secondary to a prior cancer diagnosis, e.g. radiology used in analysis of relapse or remission of a cancerous nodules

- Non-human/ animal or phantom studies
- Studies that describe AI models but do not use them in a clinical setting.
- Studies that use synthesised datasets: the radiological imaging used must be from real patients.
- fMRI studies are excluded as seen in previous AI imaging systematic reviews[7] as the computer analysis used in this data is quite separate from computer vision-based tasks.
- Reviews, commentaries, and other non-primary studies
- Single case studies

Duplicated data and multiple reports of the same study will either be removed or linked together so data is not replicated. Eligibility criteria of the study for the review will also be labelled.

**3.4 Outcomes**

***3.4.1 Primary outcome:***

The primary outcomes will consider how AI affects the performance of radiologists. ROC AUC and analysis is commonly used along with reading time, sensitivity, and specificity measurements. We are interested in comparing the difference between reader accuracy vs. reader & AI accuracy.

To be able to conduct meta-analysis, pooled mean differences in accuracy between the reader and the reader with AI will be found. An inverse-weighted meta-analysis would be used.

This is an important outcome allowing us to help us consider if AI-aided tools may prove effective for helping make risk stratification decisions for clinicians.

***3.4.2 Secondary/additional outcomes:***

Additional outcomes will reflect two main areas of interest:

- AI models and their criteria: classification, differentiation, prognostic prediction (e.g. growth rate estimation). This may also allow us to correlate what variables were most used in developing AI models and which ones were effective.
- Study design: statistical methods, completeness of data presentation and complications with analysis that may have been encountered. This will provide an important part of analysing the quality of the data that is provided.

### 3.5 Data extraction, selection & coding

The extraction covers the following broad topics: study characteristics, AI model characteristics, study outcome, methods to record study outcomes.

Study outcomes will cover:

- Diagnostic accuracy measure used. This may be in the form of:
    - ROC AUC
    - Sensitivity
    - Specificity
- Additional measures that may include:
    - Change in AUC
    - Change in sensitivity/ specificity.
    - Positive/ negative predictive values

Data will also be extracted to see if meta-analysis would be appropriate in this paper. These will include measures of uncertainty:

- Standard error
- Confidence intervals

Data for study characteristics that will be extracted will include:

- Subspeciality of imaging (neuroradiology, chest, cross sectional, MR CT, etc.)
- Type of cancer being assessed (organ and disease studied)
- Nature of the diagnostic task (e.g., characterisation, localisation, presence/absence)
- Number of readers, prior experience, training for the study
- Number of images/ patients
- Date of study (this may allow us also to see the improvement of studies across a period)

The CLAIM guideline for AI and medical imaging[12] provides a clear checklist of what data would be relevant to extract. This promotes reproducible scientific communication in AI to medical imaging papers and should be used as a 'gold-standard' for when considering studies. In accordance with this data regarding AI models that will be included will be:

- Prospective or retrospective.
- Inclusion/ exclusion criteria if apparent for participants.
- Predictive model used and targeted application of such model.

- Predictor or outcome variables apparent/ evaluation metric used.
- Whether artificial intelligence methodology used a 'custom deep learning architecture' or an established one and if so, what these were.
- How the AI assisted the radiologist: e.g. pre-screened images, identified features, probability stratification.

With regards to the multi-reader multi-case study design the following information may be extracted to provide insight into how these studies are carried out

- Study randomisation: some studies will initially start with radiologist, provide a washout period, and then do radiologist with AI. This would not cancel out any learning effects and may provide different data compared to those when studies randomise the order of the reading.
- Did the study imaging reflect clinical populations or was it modified for study purposes representing an enriched population. This may lead to context bias in the radiologist's perception.
- How were cases decided to be cancerous or non-cancerous. Was this based on single expert radiological or clinical follow-up?
- How was the risk of malignancy rated: was this a numerical categorisation or a binary option taken. For example, was malignancy risk estimated or was it based on what actions were required to be taken on a list of 6 recommendations on further actions[4]. This will form an important methodology to understand how the ROC AUC has been derived.

The data extraction sheet will be piloted with a sample with 5 included studies to confirm feasibility of the process and identify any challenges that may arise.

**3.6 Risk of bias (quality/critical assessment of included studies)**

Quality assessment remained difficult due to the study design as it remains relatively new and thus may not have an adequate critical assessment scale. Therefore, we have decided to integrate the QUADAS-2[13] with aspects from CLAIM[12] integrated to provide an adapted version that allows both the analysis of diagnostic cohort study tools as well as a checklist for the use of AI in medical imaging. Due to the scope of this task the checklist for this may be relatively simplistic but aim to provide some insight into the quality of the AI methodology used. Studies judge to be at high risk of bias will be excluded from the analysis.

### 3.7 Strategy for data synthesis (analysis plan)

We will generate a PRISMA flow diagram to summarise the number of studies retrieved and the inclusion and exclusion of studies. Summary tables will outline included study characteristics. Synthesised data will then include types of cancer and types of radiological techniques used. We will then assess the quality of evidence available and the effective use of AI in these cases and summarise findings based on the presence or absence of an effect.

***Statistical analysis***

Given that there may be heterogeneity among studies, especially given the diversity of cancerous diseases used, a meta-analysis may not be possible`. However, as the evaluated output of diagnostic accuracy for such multi-reader multi-case studies has been defined it may be possible and if the data permits, we will perform meta-analysis to combine results of similar studies using STATA version 17 software. This will be done to review test accuracy combining estimates of the sensitivity and specificity of the test from each study. A forest plot will be constructed to visualise heterogeneity and we will then assess whether any summary measures should be used.

Subgroup analysis may be beneficial based both on the study design, cancer type, speciality/ expertise (e.g. radiologist, EM, Senior, Junior doctors pulmonologists, etc.) and on the type of AI model and software used. Evaluating the AI models used and how they relate to accuracy may help us to understand which models work the best to aid doctors. A study design-related subgroup may help us to consider if study design may make a difference. This may suggest the introduction of bias effects that affect readers and thus allow us to discuss and evaluate study design. If a meta-analysis is achievable in this these can also be considered as covariates to be included in the models to investigate heterogeneity.

### 3.8: Software and data sharing

Data will be summarised using excel/google sheets to accumulate and extract all aspects of our data. The protocol will be pre-registered and all other data extraction forms shared.


## 4.0 Funding

No funding has been obtained to undertake this study.


## 5.0 Conflicts of interest

All authors declared no conflicts of interest.

## 6.0 References


1. Hosny, A., Parmar, C., Quackenbush, J., Schwartz, L. H. & Aerts, H. J. W. L. Artificial intelligence in radiology. *Nature Reviews Cancer* vol. 18 500–510 Preprint at https://doi.org/10.1038/s41568-018-0016-5 (2018).
2. Bian, Y. *et al.* Artificial Intelligence to Predict Lymph Node Metastasis at CT in Pancreatic Ductal Adenocarcinoma. *Radiology* **306**, 160–169 (2023).
3. Oren, O., Gersh, B. J. & Bhatt, D. L. Artificial intelligence in medical imaging: switching from radiographic pathological data to clinically meaningful endpoints. *Lancet Digit Health* **2**, e486–e488 (2020).
4. Kim, R. Y. *et al.* Artificial Intelligence Tool for Assessment of Indeterminate Pulmonary Nodules Detected with CT. *Radiology* **304**, 683–691 (2022).
5. Dendumrongsup, T. *et al.* Multi-Reader Multi-Case Studies Using the Area under the Receiver Operator Characteristic Curve as a Measure of Diagnostic Accuracy: Systematic Review with a Focus on Quality of Data Reporting. *PLoS One* **9**, e116018 (2014).
6. Wagner, R. F., Metz, C. E. & Campbell, G. Assessment of Medical Imaging Systems and Computer Aids: A Tutorial Review. *Acad Radiol* **14**, 723–748 (2007).
7. Kelly, B. S. *et al.* Radiology artificial intelligence: a systematic review and evaluation of methods (RAISE). *European Radiology* vol. 32 7998–8007 Preprint at https://doi.org/10.1007/s00330-022-08784-6 (2022).
8. Sadaghiani, M. S., Rowe, S. P. & Sheikhbahaei, S. Applications of artificial intelligence in oncologic 18F-FDG PET/CT imaging: a systematic review. *Ann Transl Med* **9**, 823–823 (2021).
9. Gallas, B. D. *et al.* Evaluating Imaging and Computer-aided Detection and Diagnosis Devices at the FDA. *Academic Radiology* vol. 19 463–477 Preprint at https://doi.org/10.1016/j.acra.2011.12.016 (2012).
10. Page, M. J. *et al.* The PRISMA 2020 statement: an updated guideline for reporting systematic reviews. *BMJ* n71 (2021) doi:10.1136/bmj.n71.
11. *Cochrane Handbook for Systematic Reviews of Interventions*. (Wiley, 2019). doi:10.1002/9781119536604.
12. Mongan, J., Moy, L. & Kahn, C. E. Checklist for Artificial Intelligence in Medical Imaging (CLAIM): A Guide for Authors and Reviewers. *Radiol Artif Intell* **2**, e200029 (2020).
13. Whiting, P. F. *et al.* QUADAS-2: a revised tool for the quality assessment of diagnostic accuracy studies. *Ann Intern Med* **155**, 529–36 (2011).

## Appendix A2: Search strategies

Search stragies run on 01/11/2023.

**Database: Medline (Ovid MEDLINE® Epub Ahead of Print, In-Process & Other Non-Indexed Citations, Ovid MEDLINE® Daily and Ovid MEDLINE®) 1946 to present**

**Database: Embase 1974 to Present**

1 (cancer? or tumour? or tumor? or malignancy or neoplasm* or nodule? or carcinoma? or lymphoma?).m_titl.
2 (AI or artificial intelligence or machine learning or algorithm* or computer-assisted or deep learning or radiomics or neural network?).m_titl.
3 (radio* or x-ray or pet?ct or mri or ct or magnetic resonance imaging or diagnostic imaging or image interpretation or tomograp*).m_titl.
4 (sensitivity or specificity or diagnos* or detect* or accura* or "area under curve" or observer variation or intraobserver varia* or interobserver agreement or roc or predictive value or ppv or npv or auc).ti,ab.
5 1 and 2 and 3 and 4

**Cochrane Central Register of Controlled Trials**

Title Abstract Keyword: (“Cancer*” OR “Tumo*” OR “Malignancy” OR “Neoplas*” OR “Nodule*” OR “Carcinoma” OR “Lymphoma”)
AND
Title Abstract Keyword: (“AI” OR “Artificial Intelligence” OR “Machine Learning” OR “Algorithm” OR “Computer-assisted” OR “Deep Learning” OR “Radiomics” OR “Neural Network”)
AND
Record Title: (“Radio*” OR “X-Ray” OR “PET/CT” OR “MRI” OR “Radiological Images” OR “CT” OR “Computerised Tomography” OR “Magnetic Resonance Imaging” OR “Diagnostic imaging” OR “Image interpretation” OR “tomogra*”)
AND
Title Abstract Keyword: (“Sensitivity” OR “Specificity” OR “Diagnos*” OR “Detect*” OR “Accura*” OR “Area Under Curve” OR “Observer variation” OR “Interobserver varia*” OR “Interobserver agreement” OR “Roc” OR “Predictive value” OR “PPV” OR “NPV” OR “auc”

## Appendix A3: excluded studies at full text

### Wrong Study design (not MRMC)

Park, V. Y., Lee, E., Lee, H. S., Kim, H. J., Yoon, J., Son, J., Song, K., Moon, H. J., Yoon, J. H., Kim, G. R., & Kwak, J. Y. (2021). Combining radiomics with ultrasound-based risk stratification systems for thyroid nodules: an approach for improving performance. *European Radiology*, *31*(4), 2405–2413. https://doi.org/10.1007/s00330-020-07365-9

Romeo, V., Cuocolo, R., Apolito, R., Stanzione, A., Ventimiglia, A., Vitale, A., Verde, F., Accurso, A., Amitrano, M., Insabato, L., Gencarelli, A., Buonocore, R., Argenzio, M. R., Cascone, A. M., Imbriaco, M., Maurea, S., & Brunetti, A. (2021). Clinical value of radiomics and machine learning in breast ultrasound: a multicenter study for differential diagnosis of benign and malignant lesions. *European Radiology*, *31*(12), 9511–9519. https://doi.org/10.1007/s00330-021-08009-2

Kang, F., Mu, W., Gong, J., Wang, S., Li, G., Li, G., Qin, W., Tian, J., & Wang, J. (2019). Integrating manual diagnosis into radiomics for reducing the false positive rate of 18F-FDG PET/CT diagnosis in patients with suspected lung cancer. *European Journal of Nuclear Medicine and Molecular Imaging*, *46*(13), 2770–2779. https://doi.org/10.1007/s00259-019-04418-0

Zhang, Y., Jiang, B., Zhang, L., Greuter, M. J. W., de Bock, G. H., Zhang, H., & Xie, X. (2022). Lung Nodule Detectability of Artificial Intelligence-assisted CT Image Reading in Lung Cancer Screening. *Current Medical Imaging Formerly Current Medical Imaging Reviews*, *18*(3), 327–334. https://doi.org/10.2174/1573405617666210806125953

Leibig, C., Brehmer, M., Bunk, S., Byng, D., Pinker, K., & Umutlu, L. (2022). Combining the strengths of radiologists and AI for breast cancer screening: a retrospective analysis. *The Lancet Digital Health*, *4*(7), e507–e519. https://doi.org/10.1016/S2589-7500(22)00070-X

Wang, X., You, X., Zhang, L., Huang, D., Aramini, B., Shabaturov, L., Jiang, G., & Fan, J. (2021). A radiomics model combined with XGBoost may improve the accuracy of distinguishing between mediastinal cysts and tumors: a multicenter validation analysis. *Annals of Translational Medicine*, *9*(23), 1737–1737. https://doi.org/10.21037/atm-21-5999

Bernstein, M. H., Atalay, M. K., Dibble, E. H., Maxwell, A. W. P., Karam, A. R., Agarwal, S., Ward, R. C., Healey, T. T., & Baird, G. L. (2023). Can incorrect artificial intelligence (AI) results impact radiologists, and if so, what can we do about it? A multi-reader pilot study of lung cancer detection with chest radiography. *European Radiology*, *33*(11), 8263–8269. https://doi.org/10.1007/s00330-023-09747-1

Huo, T., Xie, Y., Fang, Y., Wang, Z., Liu, P., Duan, Y., Zhang, J., Wang, H., Xue, M., Liu, S., & Ye, Z. (2023). Deep learning-based algorithm improves radiologists' performance in lung cancer bone metastases detection on computed tomography. *Frontiers in Oncology*, *13*. https://doi.org/10.3389/fonc.2023.1125637

Tam, M. D. B. S., Dyer, T., Dissez, G., Morgan, T. N., Hughes, M., Illes, J., Rasalingham, R., & Rasalingham, S. (2021). Augmenting lung cancer diagnosis on chest radiographs: positioning artificial intelligence to improve radiologist performance. *Clinical Radiology*, *76*(8), 607–614. https://doi.org/10.1016/j.crad.2021.03.021

Murchison, J. T., Ritchie, G., Senyszak, D., Nijwening, J. H., van Veenendaal, G., Wakkie, J., & van Beek, E. J. R. (2022). Validation of a deep learning computer aided system for CT based lung nodule detection, classification, and growth rate estimation in a routine clinical population. *PLOS ONE*, *17*(5), e0266799. https://doi.org/10.1371/journal.pone.0266799

Rosa, F., Martinetti, C., Magnaldi, S., Rizzo, S., Manganaro, L., Migone, S., Ardoino, S., Schettini, D., Marchiolè, P., Ragusa, T., & Gandolfo, N. (2023). Uterine mesenchymal tumors: development and preliminary results of a magnetic resonance imaging (MRI) diagnostic algorithm. *La Radiologia Medica*, *128*(7), 853–868. https://doi.org/10.1007/s11547-023-01654-1

Huang, X., Wu, Z., Zhou, A., Min, X., Qi, Q., Zhang, C., Chen, S., & Xu, P. (2021). Nomogram Combining Radiomics With the American College of Radiology Thyroid Imaging Reporting and Data System Can Improve Predictive Performance for Malignant Thyroid Nodules. *Frontiers in Oncology*, *11*. https://doi.org/10.3389/fonc.2021.737847

Sun, K., Chen, S., Zhao, J., Wang, B., Yang, Y., Wang, Y., Wu, C., & Sun, X. (2021). Convolutional Neural Network-Based Diagnostic Model for a Solid, Indeterminate Solitary Pulmonary Nodule or Mass on Computed Tomography. *Frontiers in Oncology*, *11*. https://doi.org/10.3389/fonc.2021.792062

Zhang, T., Wang, Y., Sun, Y., Yuan, M., Zhong, Y., Li, H., Yu, T., & Wang, J. (2021). High-resolution CT image analysis based on 3D convolutional neural network can enhance the classification performance of radiologists in classifying pulmonary non-solid nodules. *European Journal of Radiology*, *141*, 109810. https://doi.org/10.1016/j.ejrad.2021.109810

**Wrong study Outcome**

Nam, J. G., Hwang, E. J., Kim, J., Park, N., Lee, E. H., Kim, H. J., Nam, M., Lee, J. H., Park, C. M., & Goo, J. M. (2023). AI Improves Nodule Detection on Chest Radiographs in a Health Screening Population: A Randomized Controlled Trial. *Radiology*, *307*(2). https://doi.org/10.1148/radiol.221894

Lu, S.-L., Xiao, F.-R., Cheng, J. C.-H., Yang, W.-C., Cheng, Y.-H., Chang, Y.-C., Lin, J.-Y., Liang, C.-H., Lu, J.-T., Chen, Y.-F., & Hsu, F.-M. (2021). Randomized multi-reader evaluation of automated detection and segmentation of brain tumors in stereotactic radiosurgery with deep neural networks. *Neuro-Oncology*, *23*(9), 1560–1568. https://doi.org/10.1093/neuonc/noab071

Witowski, J., Heacock, L., Reig, B., Kang, S. K., Lewin, A., Pysarenko, K., Patel, S., Samreen, N., Rudnicki, W., Łuczyńska, E., Popiela, T., Moy, L., & Geras, K. J. (2022). Improving breast cancer diagnostics with deep learning for MRI. *Science Translational Medicine*, *14*(664). https://doi.org/10.1126/scitranslmed.abo4802

Li, M., Yang, L., Yue, Y., Xu, J., Huang, C., & Song, B. (2021). Use of Radiomics to Improve Diagnostic Performance of PI-RADS v2.1 in Prostate Cancer. *Frontiers in Oncology*, *10*. https://doi.org/10.3389/fonc.2020.631831

Li, Z., Kitajima, K., Hirata, K., Togo, R., Takenaka, J., Miyoshi, Y., Kudo, K., Ogawa, T., & Haseyama, M. (2021). Preliminary study of AI-assisted diagnosis using FDG-PET/CT for axillary lymph node metastasis in patients with breast cancer. *EJNMMI Research*, *11*(1), 10. https://doi.org/10.1186/s13550-021-00751-4

Sadik, M., López-Urdaneta, J., Ulén, J., Enqvist, O., Andersson, P.-O., Kumar, R., & Trägårdh, E. (2023). Artificial Intelligence Increases the Agreement among Physicians Classifying Focal Skeleton/Bone Marrow Uptake in Hodgkin's Lymphoma Patients Staged with [18F]FDG PET/CT—a Retrospective Study. *Nuclear Medicine and Molecular Imaging*, *57*(2), 110–116. https://doi.org/10.1007/s13139-022-00765-3

**Study Not Completed**

Baldwin, D., Woznitza, N., Lee, R., Navani, N., Nair, A., & Srivastava, S. (2022). EP01.06-002 Impact of Immediate AI Enabled Patient Triage to Chest CT on the Lung Cancer Pathway: LungIMPACT. *Journal of Thoracic Oncology*, *17*(9), S187. https://doi.org/10.1016/j.jtho.2022.07.314

KCT0005051. (2022). *Diagnosis of lung nodule and lung cancer on screening chest radiographs: comparative clinical trial for evaluation of artificial intelligence-integrated PACS versus conventional PACS*.
NCT05968898. (2023). *Radiomics Tool for Pulmonary Nodule Risk Stratification*.

**Conference Abstracts**

Lee, J. H., Hong, H., Nam, G., Hwang, E. J., & Park, C. M. (2023). Effect of Human-AI Interaction on Detection of Malignant Lung Nodules on Chest Radiographs. *Radiology*, *307*(5). https://doi.org/10.1148/radiol.222976
Ajmera, P., Kharat, A., Kulkarni, V., Kathuria, S., Saini, M., Patil, S., Raj, P., Ghuwalewala, S., Edara, M., Kapoor, R., Goyal, S., M., K., Singh, T., M., S., Rathi, S., Seth, J., & Pant, R. (2022). Evaluating the effectivity of deploying a deep learning-based algorithm to aid clinicians and radiologists in diagnosing pulmonary nodules on chest radiographs. *11.01 - Lung Cancer*, 4308. https://doi.org/10.1183/13993003.congress-2022.4308
TAKAISHI, T. (2022). THE USE OF ARTIFICIAL INTELLIGENCE SYSTEM TO DETECT LUNG NODULES ON CT SCAN IN REAL CLINICAL SETTINGS. *Chest*, *161*(6), A319. https://doi.org/10.1016/j.chest.2021.12.349

**Duplicates**

Yasaka, K., Hatano, S., Mizuki, M., Okimoto, N., Kubo, T., Shibata, E., Watadani, T., & Abe, O. (2023). Effects of deep learning on radiologists' and radiology residents' performance in identifying esophageal cancer on CT. *The British Journal of Radiology*, *96*(1150). https://doi.org/10.1
